# A Post-Quantum Sensor-to-Blockchain Transaction Framework with CRQC-Aware Exposure Minimization for Next-Generation Sensor Networks

**DOI:** 10.3390/s26144327

**Published:** 2026-07-08

**Authors:** Bora Bugra Sezer

**Affiliations:** Graduate School of Natural and Applied Sciences, Ege University, İzmir 35040, Türkiye; bora.bugra.sezer@ege.edu.tr

**Keywords:** post-quantum blockchain, sensor networks, CRQC-aware security, threshold authorization, quantum exposure score

## Abstract

Blockchain-based sensor networks rely on public-key cryptography for transaction verification, auditability, and data integrity. However, widely used public-key mechanisms are quantum-vulnerable in the presence of Cryptographically Relevant Quantum Computers (CRQCs), requiring sensor-to-blockchain transactions to address both post-quantum security and exposure control. This paper proposes a post-quantum sensor-to-blockchain transaction framework that minimizes CRQC-aware exposure while preserving low-cost auditability. It defines a transaction workflow that represents sensor data through hash-based commitments instead of storing raw measurements on-chain. The workflow combines Module-Lattice-Based Digital Signature Algorithm (ML-DSA)-based authentication, threshold-based authorization, Module-Lattice-Based Key Encapsulation Mechanism (ML-KEM)-protected relay communication, and an event-based smart contract (EBSC) for compact audit recording. A Quantum Exposure Score (QES) is introduced as a transaction-level metric to quantify CRQC-induced exposure across cryptographic, relay, key-lifecycle, migration-readiness, and authorization dimensions. The framework is evaluated using differential pulse voltammetry (DPV) electrochemical sensor data, Constrained Application Protocol (CoAP) communication, and a Ganache-based blockchain, with scalability runs of up to 10,000 sensor transactions and ablation baselines. Compared with full on-chain storage, EBSC reduces gas consumption by approximately 80%, while QES decreases from 100 in the classical open scenario to 4 in the full framework. These results demonstrate that the proposed design provides a practical path for post-quantum secure sensor-to-blockchain transactions.

## 1. Introduction

Next-generation sensor networks (NSNs) continuously generate data across health monitoring, environmental monitoring, industrial automation, smart cities, and biosensor-based analysis. In these systems, sensor nodes, edge gateways, and blockchain-based audit layers are used together to ensure data integrity, traceability, immutability, and verifiability. The blockchain has become a powerful security component for sensor networks because it reduces reliance on centralized trust authorities, makes it difficult to alter records retrospectively, and provides automated auditing through smart contracts [1,2,3,4]. However, many blockchain-based sensor network architectures still rely on classical public-key cryptography for transaction signing, device authentication, and smart contract interaction. In particular, elliptic-curve-based signature mechanisms such as ECDSA, Schnorr, and others are widely used in transaction verification, device authentication, validator authorization, and smart contract interactions. The security of these structures relies on the assumption that the elliptic-curve discrete logarithm problem (ECDLP) is computationally infeasible to solve on classical computers. However, Shor’s algorithm can overcome this assumption using a sufficiently large, fault-tolerant quantum computer [5]. In this context, the concept of a Cryptographically Relevant Quantum Computer (CRQC) is particularly important. CRQC refers to the quantum computing capability that can threaten common public-key structures such as RSA, ECC, ECDSA, Schnorr, or BLS on a practical scale. Recent resource estimates show that Shor-based attacks on 256-bit elliptic curves commonly used in blockchains, such as secp256k1, can be compiled with lower logical qubit and Toffoli gate costs than previous estimates. In particular, current estimates for secp256k1 indicate that Shor-based attacks against 256-bit ECDLP can be implemented with approximately 1200 logical qubits and 90 million Toffoli gates, or 1450 logical qubits and 70 million Toffoli gates [6]. This result makes public-key exposure, relay/mempool visibility, and transaction propagation time first-class security parameters in blockchain systems.

The NIST-conducted transition to post-quantum cryptography also supports this need. NIST IR 8547 emphasizes that the publication of post-quantum standards is only the first stage, and that migrating existing systems to these standards requires a planned, long-term process [7]. In the same process, ML-KEM was standardized with FIPS 203 for its key encapsulation mechanism, and ML-DSA was standardized with FIPS 204 as a digital signature standard [8,9]. These standards demonstrate that post-quantum security in IoT and sensor networks with long lifecycles is not only a future-proof option but also an engineering requirement that must be planned at the protocol level.

This observation points to a critical design problem for sensor networks. When sensor data or a sensor event is sent to the blockchain, the transaction object, public key, signature, device identity, relay information, and timestamp can become visible at different layers. The public mempool, public relay, reused keys, long-lived device identities, and single gateway signers create a measurable attack surface for CRQC-capable attackers. Therefore, post-quantum security in sensor networks requires a design that addresses transaction flow, public-key exposure, relay/mempool visibility, threshold authorization, key lifecycle, and on-chain audit overhead together. Current studies demonstrate that post-quantum signatures can be integrated into blockchain transactions, that signature schemes such as Dilithium/ML-DSA and Falcon can be used in transaction validation, and that post-quantum signatures are feasible in low-cost IoT devices [1,2]. Edge-based approaches reduce the computational load on edge devices by offloading heavy post-quantum validation operations to resource-intensive edge servers [3]. Furthermore, permissioned blockchain, IPFS, smart contract-based batch verification, and zero-trust IoT approaches highlight the importance of engineering metrics such as latency, throughput, and storage overhead in blockchain-based IoT systems [4]. However, these studies do not provide a transaction-level security model that quantifies the public-key exposure and relay/mempool visibility of sensor network–blockchain transactions against CRQC-capable attackers.

This study proposes a CRQC-aware post-quantum-supported blockchain transaction security framework for next-generation sensor networks. The proposed framework consists of a sensor node, an edge gateway, a threshold authorization layer, a protected relay, and an event-based smart contract (EBSC). The main objective is to reduce public-key exposure, relay/mempool visibility, single-signer dependency, and on-chain audit overhead in the transaction flow that transfers sensor data to the blockchain. To validate the feasibility of the proposed framework, DPV electrochemical sensor data obtained from the experimental study in [10,11] are processed through Constrained Application Protocol (CoAP)-based sensor communication [12], a Node.js edge gateway, PQClean/WebAssembly (WASM)-based post-quantum cryptographic operations, and an EBSC deployed on Ganache. Experimental evaluation is conducted based on data transmission time, post-quantum processing time, commitment generation cost, event-based smart contract gas consumption, and on-chain overhead difference compared to a storage-based approach.

### Motivation and Contributions

Under a CRQC-capable adversary, sensor-to-blockchain transaction security is determined by the selected signature mechanism, public-key visibility, relay/mempool exposure, key reuse, and the key lifecycle state across the transaction flow. Public-key visibility level, relay/mempool exposure duration, single-gateway or single-signer dependency, key reuse, and key lifecycle state are all elements that directly define the attack surface. Therefore, in next-generation sensor networks, post-quantum security requires a broader transaction security model than simply modifying the transaction signature. Current approaches are making significant progress in areas such as post-quantum signature integration, PQC feasibility on IoT devices, edge-assisted verification, and smart contract-based recording/verification. However, these approaches do not evaluate public-key visibility, relay/mempool exposure duration, single-signer dependency, key reuse, and post-quantum migration readiness under a common transaction-level model. This gap necessitates addressing public-key exposure and transaction propagation visibility as measurable security parameters in sensor network–blockchain transactions against CRQC-capable attackers. The research objective of this study is to quantify the CRQC-induced attack surface in the transaction flow where sensor data is transferred to the blockchain, as well as to minimize this attack surface using post-quantum authentication, threshold authorization, protected relay, key lifecycle control, and EBSC.

The main contributions of this study are summarized as follows:

1. We propose a transaction-level threat model for next-generation sensor networks against CRQC-capable attackers. This model considers public-key exposure, relay/mempool visibility, key reuse, single-signer dependency, long-lived sensor identities, and post-quantum migration readiness.
2. We formulate a quantitative risk metric, the Quantum Exposure Score (QES), for sensor network–blockchain transactions. QES evaluates public-key visibility level, relay/mempool exposure duration, quantum vulnerability of the signature mechanism, threshold authorization status, key reuse, and key lifecycle state together.
3. We design a transaction workflow that combines threshold-based transaction authorization, hash-based commitment, controlled reveal, ML-KEM-based protected relay communication, and edge-layer ML-DSA-based post-quantum authentication for recording sensor data commitments at the blockchain layer.
4. We implement an event-based smart contract (EBSC) to reduce blockchain overhead and communication costs at the blockchain layer.
5. We evaluate the proposed framework by using CoAP-based sensor communication, PQClean/WASM-based post-quantum execution, a Node.js edge gateway, and a Ganache-based local Ethereum-compatible blockchain environment on real DPV electrochemical sensor data.

## 2. Literature Review

Post-quantum blockchain security has become a fundamental area of research for the long-term security of blockchain systems relying on classical public-key cryptography. Yang et al. stated that post-quantum blockchains aim to strengthen existing blockchain architectures with quantum-resistant classical cryptographic primitives [13]. Gharavi et al. addressed post-quantum blockchain security in the context of IoT and showed that PQC primitives become critical for secure data sharing, authentication, and blockchain-based security in IoT/sensor systems with long lifecycles [14]. These survey studies illustrate the general direction of the field. However, they do not offer a transaction-level risk model that measures public-key exposure, relay/mempool visibility, and key lifecycle status across the sensor-to-blockchain transaction workflow, in which sensor data is transferred directly to the blockchain. One of the studies that addresses quantum resilience in blockchain networks in a more applied way is presented by Allende et al. Their study discusses second-signature, post-quantum migration strategies, as well as network-level compatibility issues, to make existing blockchain networks quantum-resilient [15]. Puneyani and Bhat proposed a CRYSTALS–Dilithium-based approach for transaction signing and verification in quantum-resilient blockchain protocols [1]. In their study, Dilithium is used as a digital signature component for blockchain transactions, whereas Kyber is considered more suitable for key exchange than for transaction signing. This distinction supports the use of ML-KEM for key establishment in protected relay communication and ML-DSA for transaction authentication in the proposed study.

The applicability of PQC algorithms to IoT and sensor systems is further investigated due to resource constraints, energy consumption, memory requirements, and processing latency. Castiglione et al. implemented a Dilithium-5 application on an ESP32 microcontroller to secure low-cost IoT devices with post-quantum blockchain systems [2]. Their study aimed to enable autonomous transaction submission from the IoT device to the post-quantum-ready blockchain and presents a use case using health sensor data. Edge-based studies offer a significant solution direction for reducing heavy cryptographic operations in resource-constrained devices. Hao and Wu proposed a PUF-enabled approach for the efficient verification of post-quantum blockchain transactions in edge/IoT environments [3]. In their study, heavy post-quantum verification operations are offloaded to resource-rich edge servers, while edge devices are limited to lighter tasks. Similarly, studies on PQ signature validation and implementation costs in constrained device environments show that post-quantum algorithms, despite their security advantage, need to be carefully positioned with respect to signature size, memory usage, and processing time [16,17,18]. Based on these findings, the proposed study uses the edge gateway not only as a layer that reduces computational overhead but also as a transaction security layer that provides public-key exposure, threshold authorization, and protected relay control.

Zero-trust architectures, IPFS-based off-chain storage, anomaly detection, and smart contract-based mechanisms have also been widely investigated for strengthening blockchain-enabled IoT security. Alanazi et al. proposed a framework that combines a permissioned blockchain, post-quantum cryptography, IPFS-based off-chain storage, and anomaly detection components for zero-trust IoT environments [4]. Their study demonstrates the need for empirical analysis of IoT-blockchain systems by jointly evaluating metrics such as latency, throughput, storage overhead, and anomaly-detection accuracy. However, the main focus of their approach is zero-trust IoT security and anomaly detection, and it thus does not model transaction propagation visibility, public-key exposure, or relay/mempool exposure time as security metrics against a CRQC-capable attacker. Lightweight blockchain- and smart contract-based auditing studies are directly important for reducing on-chain overhead in sensor networks. Novo proposed a blockchain-based architecture for scalable access management in IoT ecosystems [19]. Liu et al. proposed LightChain, a lightweight blockchain structure for industrial IoT environments that aims to reduce computation, storage, and network usage [20]. Garba proposed LightCERT4IoTs, a blockchain-based lightweight certificate authentication scheme for IoT applications [21]. These studies show that when using blockchain in IoT/sensor networks, not only security but also blockchain overhead, communication costs, storage overhead, and authentication costs should be considered. However, these approaches do not combine the low-overhead goal with CRQC-aware exposure modeling, relay/mempool visibility, or post-quantum transaction authentication. Smart contracts are widely used in blockchain-based IoT and sensor systems for data integrity, auditability, access control, batch verification, and settlement processes.

The current literature shows that post-quantum signatures can be integrated into blockchain systems, PQC schemes can be implemented on IoT devices, computational overhead can be reduced through edge-assisted verification, and data validation and batch settlement can be supported by smart contract-based approaches. However, the transaction-level exposure model, which holistically addresses the transaction flow where sensor data is transferred to the blockchain against CRQC-capable attackers in next-generation sensor networks, is not yet sufficiently mature. This situation manifests itself in four key points. First, current post-quantum blockchain studies primarily focus on changes to signature algorithms and transaction verification costs. Studies that quantitatively evaluate how long the public key remains visible in the relay, mempool, or on-chain layer are limited. Second, IoT and edge-based approaches utilize offloading, PUF, or microcontroller-level PQC implementations to reduce computational load. However, single-gateway or single-signatory dependency is often not modeled as a direct component of CRQC-aware transaction risk. Third, lightweight blockchain studies focus on reducing storage and network overhead. However, these optimizations are not considered alongside quantum-related transaction security parameters such as public-key exposure, relay visibility, and key lifecycle. Fourth, smart contract-based approaches are generally used for data integrity, batch verification, and settlement purposes. Designing the EBSC layer as a low-cost audit layer that publishes exposure-aware metadata can be considered a separate research direction. This study proposes a CRQC-aware, exposure-minimizing, and threshold-authorized blockchain transaction framework that integrates these research directions. Table 1 summarizes the qualitative comparison between representative research studies and the proposed framework.

## 3. Preliminaries

This section introduces the mathematical notation and lattice-based cryptographic background used in the proposed framework. In particular, it summarizes the module-lattice setting, ML-KEM, and ML-DSA as quantum-safe cryptographic primitives that support the transition from classical public-key infrastructures to post-quantum-secure transaction authentication and key establishment. Table 2 summarizes the main notation used throughout this paper.

### 3.1. Algebraic Notation and Module-Lattice Setting

In this study, *q* denotes the prime or an appropriately chosen modulus, n denotes the polynomial degree, *k* denotes the module rank, and λ denotes the security parameter. $Z_{q}$ denotes the ring-of-integers modulo *q*. In lattice-based KEM and signature schemes, operations are generally defined on the polynomial ring $R_{q}=Z_{q}\left[x\right]/\left(x^{n}+1\right)$ [22].

This structure consists of polynomials whose coefficients are defined over $Z_{q}$ and are reduced to modulo $x^{n}+1$. In module-lattice-based schemes such as ML-KEM and ML-DSA, polynomial vectors are processed on $R_{q}^{k}$ and polynomial matrices are processed on $R_{q}^{k\times k}$. This structure both connects security assumptions to module-lattice problems and enables efficient polynomial multiplication using the Number Theoretic Transform (NTT) [23].

Let a polynomial $a\left(x\right)=a_{0}+a_{1}x+\ldots +a_{n-1}x^{n-1}\in R_{q}$. The multiplication of two polynomials is calculated as modulo $x^{n}+1$ and modulo *q*. That is, it is expressed as $c\left(x\right)=a\left(x\right)\cdot b\left(x\right)\;\mathrm{mod}\;\left(x^{n}+1,q\right)$.

Using the schoolbook multiplication method, this multiplication is calculated with $O(n^{2})$ complexity. When using NTT, polynomials are transformed to the NTT space, enabling more efficient multiplication. This operation can be expressed as $a\cdot b=\mathrm{INTT}\left(\mathrm{NTT}(a)\circ \mathrm{NTT}(b)\right)$, where ∘ represents pointwise multiplication. NTT-based multiplication is a key performance component in ML-KEM and ML-DSA implementations because it enables polynomial multiplication with O(nlogn) complexity [22,23]. While pointwise multiplication can be implemented over full NTT layers in ML-DSA, some multiplications are treated as pair-pointwise multiplication due to the ring and root structure used in ML-KEM [23]. This distinction is important for performance, memory access, and optimization strategies in ML-KEM and ML-DSA implementations.

### 3.2. Learning with Errors and Module-LWE

Learning With Errors (LWE) is one of the fundamental difficulty assumptions of lattice-based cryptography. Introduced by Regev, the LWE problem is considered one of the most important problems forming the security basis of modern lattice-based encryption and key-establishment mechanisms [24]. In simple terms, the LWE problem assumes it is difficult to distinguish between two distributions. The first distribution is (A,A·s+e) and the second distribution is (A,u), where *A* is a random matrix, *s* is a secret vector, *e* is an error vector, and *u* is a uniform random vector. The difficulty of LWE stems from the error term *e*, which prevents linear algebraic analysis [22,24].

Module Learning With Errors (MLWE) generalizes the LWE problem onto polynomial rings and module-lattice structures. In MLWE, examples are usually of the form $\left(A,A\cdot s+e\right)\in R_{q}^{k\times k}\times R_{q}^{k}$, where *A* is a random polynomial matrix over $R_{q}^{k\times k}$, *s* is a hidden polynomial vector with small coefficients, *e* is an error polynomial vector, and χ is a small-error distribution. The MLWE problem renders difficult to distinguish the distribution (A,A·s+e) from the uniform distribution (A,u). This indistinguishability is expressed in terms of computational indistinguishability [22,23,25]. MLWE is the main security assumption underlying ML-KEM. In contrast, ML-DSA relies on module-lattice hardness assumptions, mainly related to the Module Short Integer Solution (MSIS) problem and its variants [9,26]. Therefore, MLWE and MSIS are essential for understanding the cryptographic security foundation of the post-quantum building blocks used within the proposed framework.

### 3.3. Module-Lattice-Based Key Encapsulation Mechanism

A key encapsulation mechanism (KEM) enables two parties to establish a shared symmetric session key over a public or otherwise untrusted channel. ML-KEM is a module-lattice-based KEM standardized under NIST FIPS 203 and is based on the CRYSTALS–Kyber design [8]. Its security is built on the MLWE problem over the ring $R_{q}=Z_{q}\left[x\right]/\left(x^{256}+1\right)$, where n=256 and q=3329. The module rank *k* varies according to the selected security level, resulting in the ML-KEM-512, ML-KEM-768, and ML-KEM-1024 parameter sets. In ML-KEM, a Fujisaki–Okamoto-type transformation is applied to an underlying IND-CPA-secure module-lattice-based public-key encryption (PKE) scheme to obtain an IND-CCA2-secure KEM [22,23,25]. The construction can be summarized as follows [22]:

(1) $$\begin{array}{cc} \mathrm{KEM.}\;\mathrm{KeyGen}:\;(pk,sk) & \leftarrow \mathrm{CPA.}\;\mathrm{KeyGen},\\ h & \leftarrow G(pk),\\ z & \leftarrow {\left\{0,1\right\}}^{256},\\ dk & \leftarrow (sk,pk,h,z). \end{array}$$

(2) $$\begin{array}{cc} \mathrm{KEM.}\;\mathrm{Encaps}(pk):\;h & \leftarrow G(pk),\\ m & \leftarrow {\left\{0,1\right\}}^{256},\\ \left(K,\rho \right) & \leftarrow H(m,h),\\ ct & \leftarrow \mathrm{CPA.}\;\mathrm{Enc}(pk,m;\rho ),\\ \; & \mathrm{return}(ct,K). \end{array}$$

(3) $$\begin{array}{cc} \mathrm{KEM.}\;\mathrm{Decaps}(dk,ct):\;(sk,pk,h,z) & \leftarrow dk,\\ m\prime & \leftarrow \mathrm{CPA.}\;\mathrm{Dec}(sk,ct),\\ \left(K\prime ,\rho \prime \right) & \leftarrow H(m\prime ,h),\\ ct\prime & \leftarrow \mathrm{CPA.}\;\mathrm{Enc}(pk,m\prime ;\rho \prime ),\\ K_{\mathrm{out}} & =\left\{\begin{array}{cc} K\prime , & \mathrm{if}\;ct=ct\prime ,\\ H(z,ct), & \mathrm{otherwise}. \end{array}\right. \end{array}$$

where pk and sk denote the public and secret key material generated by the underlying CPA-secure encryption component, h=G(pk) is used in key derivation, and $z\in {\left\{0,1\right\}}^{256}$ is secret random material used for implicit rejection. In this high-level representation, dk=(sk,pk,h,z) denotes the decapsulation key. The encapsulation algorithm samples a 256-bit message *m*, derives the shared key *K* and encryption randomness ρ, and outputs the ciphertext ct. During decapsulation, m′ is recovered from ct, (K′,ρ′) is recomputed, and ct′ is regenerated for consistency checking. If ct=ct′, the output key is K′; otherwise, H(z,ct) is returned as fallback pseudorandom key material. In the underlying PKE component, the public key is derived from the noisy module-linear relation t=A·s+e, where *A* is a polynomial matrix expanded from a public seed, *s* is a small secret polynomial vector, and *e* is an error polynomial vector.

In this study, ML-KEM-512 is selected for protected relay session key establishment because it provides the lowest computational and communication overhead among the standardized ML-KEM parameter sets while still offering NIST security category 1. The ML-KEM aims to establish a post-quantum secure session key between an edge gateway and a protected relay.

### 3.4. Module-Lattice-Based Digital Signature Algorithm

A digital signature scheme is used to verify the source of a message (authentication), ensure its integrity, and provide non-repudiation. ML-DSA is a module-lattice-based digital signature standard standardized under NIST FIPS 204 [9]. It is based on the CRYSTALS–Dilithium design. The security of ML-DSA is related to the difficulty of MLWE and MSIS derivative problems [26]. Furthermore, ML-DSA relies on a structure converted from a lattice-based Σ-protocol to a signature scheme using the Fiat–Shamir-with-aborts approach [23,27]. The high-level ML-DSA signing and verification flow can be summarized as follows [22]:

(4) $$\begin{array}{cc} \mathrm{ML-DSA.}\;\mathrm{KeyGen}:\;A & \leftarrow \mathrm{ExpandA}(\rho ),\\ \left(s_{1},s_{2}\right) & \leftarrow S_{\eta },\\ t & =As_{1}+s_{2},\\ \left(t_{1},t_{0}\right) & \leftarrow \mathrm{Power2Round}(t),\\ pk & \leftarrow (\rho ,t_{1}),\\ sk & \leftarrow (\rho ,s_{1},s_{2},t_{0},pk). \end{array}$$

(5) $$\begin{array}{cc} \mathrm{ML-DSA.}\;\mathrm{Sign}(sk,m):\;\mu & \leftarrow H(pk,m),\\ y & \leftarrow S_{\gamma_{1}},\\ w & =Ay,\\ c & \leftarrow H(\mu ,\mathrm{HighBits}(w)),\\ z & =y+cs_{1},\\ h & \leftarrow \mathrm{Hint}(w,c,s_{2},t_{0}),\\ \sigma & \leftarrow (z,c,h), \end{array}$$

where the signing procedure is restarted if the norm bounds for *z* or the hint-generation checks fail.

(6) $$\begin{array}{cc} \mathrm{ML-DSA.}\;\mathrm{Verify}(pk,m,\sigma ):\;\mathrm{parse}\;\sigma & =(z,c,h),\\ \mu & \leftarrow H(pk,m),\\ {\tilde{w}}_{1} & \leftarrow \mathrm{UseHint}(h,Az-c\;\mathrm{Reconstruct}\left(t_{1}\right)),\\ c\prime & \leftarrow H(\mu ,{\tilde{w}}_{1}),\\ b & =\left\{\begin{array}{cc} 1, & \mathrm{if}\;c\prime =c\;\mathrm{and}\;z\;\mathrm{satisfies}\;\mathrm{the}\;\mathrm{norm}\;\mathrm{bound},\\ 0, & \mathrm{otherwise}. \end{array}\right. \end{array}$$

where *A* is the public polynomial matrix expanded from the seed ρ, $s_{1}$ and $s_{2}$ are short secret polynomial vectors, and $t=As_{1}+s_{2}$ is the noisy module-linear relation used to derive the public key. The values $t_{1}$ and $t_{0}$ denote the high- and low-order parts of *t*, respectively. During signing, *y* acts as a masking vector, *c* is the Fiat–Shamir challenge, *z* is the response vector, and *h* is the hint information used during verification. The rejection step prevents the response vector from leaking information about the secret key, while verification checks whether the challenge reconstructed from the message, public key, and signature components is consistent with the received challenge. The ML-DSA operations are executed in the ring $R_{q}=Z_{q}\left[x\right]/\left(x^{256}+1\right)$, and *q* = 8,380,417 is used. The standard security levels are defined as ML-DSA-44, ML-DSA-65, and ML-DSA-87 [9]. ML-DSA heavily utilizes extendable-output-function (XOF) structures such as SHAKE128 and SHAKE256 [28].

In this study, ML-DSA-44 is selected for post-quantum transaction authentication because it is the most lightweight standardized ML-DSA parameter set and provides post-quantum digital signature security.

## 4. System and Threat Model

This section defines the system and threat models for the proposed CRQC-aware blockchain transaction security framework. The system model describes how sensor data are processed by the edge gateway and converted into a post-quantum-aware, exposure-minimizing blockchain transaction flow. The threat model identifies which visibility points CRQC-capable adversaries can exploit in sensor network–blockchain transactions and which risks the system aims to mitigate.

### 4.1. System Model

The proposed system aims to securely and cost-effectively transfer data generated in next-generation sensor networks to the blockchain-based layer with post-quantum awareness. The system consists of sensor nodes, an edge gateway, authorizing edge nodes, a protected relay, a blockchain network, and an EBSC component. The sensor node $S_{i}$ is the source unit that generates data from physical, biomedical, industrial, or environmental sources. In the experimental context of this study, the sensor data $d_{i}$ represents DPV electrochemical sensor data. The sensor nodes are considered resource-constrained. The edge gateway is the middleware that transforms the sensor data into a blockchain transaction object. The edge gateway *G* receives data from the sensors, generates the data hash, produces the public-key hash, calculates the commitment value, executes the ML-DSA-based transaction authentication process, calculates the QES value, and initiates the threshold authorization process for the transaction. Post-quantum signing, relay protection, exposure assessment, and blockchain transaction preparation tasks are performed at the edge layer. Authorizing edge nodes $V_{j}$ ensures that the transaction commitment is validated before being transferred to the protected relay and EBSC layers. Each $V_{j}$ generates a confirmation bit $a_{j}$ for the commitment. When at least *t* authorizing node confirmations are provided, $Auth_{i}=1$, and the transaction is transferred to the next stage. This mechanism is used to reduce single-gateway or single-signer dependency. Protected relay *R* is used to reduce the direct visible transfer of transaction metadata to the public mempool or open relay layer. A post-quantum secure session key (KR) is established between the edge gateway and the protected relay using ML-KEM-512. This session key ensures the protection of transaction metadata with AEAD. The blockchain network is the distributed ledger layer where transaction records are kept in an auditable manner. It uses EBSC to reduce on-chain storage overhead, gas costs, and communication costs.

The flow of transferring sensor data to the blockchain layer in the proposed system can be summarized as follows:
Sensor Node → CoAP → Edge Gateway → Threshold Authorization→ ML-KEM Protected Relay → EBSC

In this flow, the sensor node first generates the data Si and di and transmits it to the edge gateway via CoAP. The edge gateway generates the values $h_{i}=H\left(d_{i}\;\|\;ts_{i}\right)$ and $pkHash_{i}=H\left(pk_{i}\right)$. Then, the commitment value $com_{i}=H(h_{i}\;\|\;pkHash_{i}\;\|\;nonce_{i}\;\|\;kv_{i}\;\|\;policy_{i})$ is calculated. The transaction metadata is created in the form $m_{i}=com_{i}\|kv_{i}\|Auth_{i}\left\|relayMode_{i}\right\|ts_{i}$ and signed with ML-DSA. When the threshold authorization condition is met, the transaction is transferred to the protected relay layer. A session key based on ML-KEM-512 is established with the relay, and the transaction metadata is protected with this key. In the final stage, EBSC publishes the auditable metadata as an event.

### 4.2. Transaction Lifecycle

In the proposed framework, the lifecycle of a sensor-to-blockchain transaction comprises six stages.

1. The first stage is data generation and transmission. The sensor node Si generates the data di and sends it to the edge gateway via CoAP.
2. The second stage is commitment generation. The edge gateway generates the hi value from the sensor data, the $pkHash_{i}$ value from the public key, and the $com_{i}$ value with the relevant nonce, key version, and policy information. This stage creates a verifiable, low-dimensional representation of the raw sensor data rather than storing it on the chain.
3. The third stage is post-quantum authentication. The edge gateway or authorized signer generates an ML-DSA signature on the message $m_{i}=com_{i}\;\|\;kv_{i}\;\|\;Auth_{i}\;\|\;relayMode_{i}\;\|\;ts_{i}$.
4. The fourth stage is threshold authorization. The authorizing edge nodes $V_{j}$ generate the acknowledgment bit for $com_{i}$. $Auth_{i}$ becomes 1 when at least *t* confirmations are received. If Authi = 0, the transaction is not transmitted to the protected relay and EBSC layers.
5. The fifth stage is protected relay transmission. The KR is established between the edge gateway and the protected relay using ML-KEM-512. The transaction metadata is encrypted with AEAD and transmitted to the relay layer.
6. The sixth stage is the event-based blockchain stage. EBSC publishes the values $com_{i}$, $kv_{i}$, $Auth_{i}$, $relayMode_{i}$, $qes_{i}$, and $ts_{i}$ as events. These events create an auditable record of the transaction without storing raw data or large cryptographic outputs on the chain.

### 4.3. Threat Model

This study defines a threat model considering a CRQC-capable adversary. The attacker can observe public key information, signature metadata, relay/mempool visibility, key version information, transaction propagation time, and on-chain event logs that emerge throughout the sensor network–blockchain transaction flow. The attacker’s goal is to use these visibility points to escalate risks, including transaction linkage, key exposure, signer targeting, relay observation, and future CRQC-based private key recovery. It is not assumed that the attacker directly obtains all sensor data or all private keys. Nor is it assumed that the attacker completely controls the blockchain consensus or has compromised all edge gateways. Instead, it is assumed that the attacker can gather information through the public mempool, public relays, on-chain event logs, and reused public keys. This model specifically targets scenarios where CRQC resource estimates make public-key visibility and transaction propagation time more critical from a security perspective.

The attacker’s primary capabilities are defined as follows:

1. **Public-key observation:** The attacker can observe the public key, public-key hash, signature verification information, or key version information during transaction propagation. Prolonged visibility of the public key can create an attack window usable for private key extraction under CRQC.
2. **Relay/mempool monitoring:** The attacker can monitor transaction propagation in the public mempool or public relay layer. This monitoring can reveal when the transaction was created, which key version is associated with it, which relay mode it was transported through, and how long it remained observable.
3. **Key reuse exploitation:** The attacker can monitor the reuse of the same public key or the same device ID in multiple transactions. Key reuse increases the risk of transaction linkage, profiling, and exposure under CRQC.
4. **Single-signer targeting:** An attacker may target a node where transactions are validated by a single gateway or single signer. Single-signer dependency creates a central breaking point in the transaction generation and validation process.
5. **Audit metadata analysis:** An attacker can analyze the event metadata published by the EBSC. If event logs contain excessive information, the risk of transaction linking and profiling may increase. Therefore, the EBSC is designed to publish only limited metadata such as commitment, key version, threshold status, relay mode, QES level, and timestamp.

### 4.4. Exposure-Oriented Attack Settings

The proposed threat model addresses five exposure-oriented attack scenarios regarding CRQC-aware transaction security.

1. **At-rest exposure:** This occurs when the public key, long-lived device ID, or transaction-related metadata is persistently visible in historical chain records. This allows an attacker with a sufficient CRQC capability to target historical records in the future.
2. **On-spend exposure:** After a transaction is signed, it becomes visible at the relay or mempool layer. An attacker can observe the public key, signature, and transaction metadata within this visibility window. CRQC source predictions make this visibility window a parameter that must be directly considered for transaction security.
3. **Relay exposure:** If transaction metadata is transmitted over an unprotected relay layer, an attacker can observe transaction-related fields such as commitment, signature, key version, relay mode, or QES. A protected relay is used to reduce this visibility.
4. **Signer-centralization exposure:** Authorization of a transaction by a single gateway or single signer creates a central target for the attacker. Threshold authorization is used to mitigate this risk.
5. **Audit metadata exposure:** Records published via smart contracts containing excessive information can increase the risk of transaction linking and profiling. Therefore, EBSC publishes only limited, verifiable audit metadata, not raw sensor data or large cryptographic outputs.

This threat model identifies at which exposure points the post-quantum building blocks are positioned to mitigate in the sensor network–blockchain process flow. This structure is designed to jointly reduce public-key exposure, relay/mempool visibility, single-signer dependency, and on-chain overhead.

### 4.5. Trust Assumptions and Compromise Scope

The proposed trust model defines the assumptions under which each entity in the transaction workflow is considered reliable and specifies which security properties remain available when a component is observed or partially compromised. In this scope, the model considers that an adversary may observe public transaction metadata and may target selected workflow components. Complete blockchain-consensus compromise and simultaneous compromise of all edge-side entities are outside the evaluated scope. Table 3 summarizes the trust assumption, compromise impact, and remaining security properties for each component.

## 5. Proposed Framework

**Figure 1:**
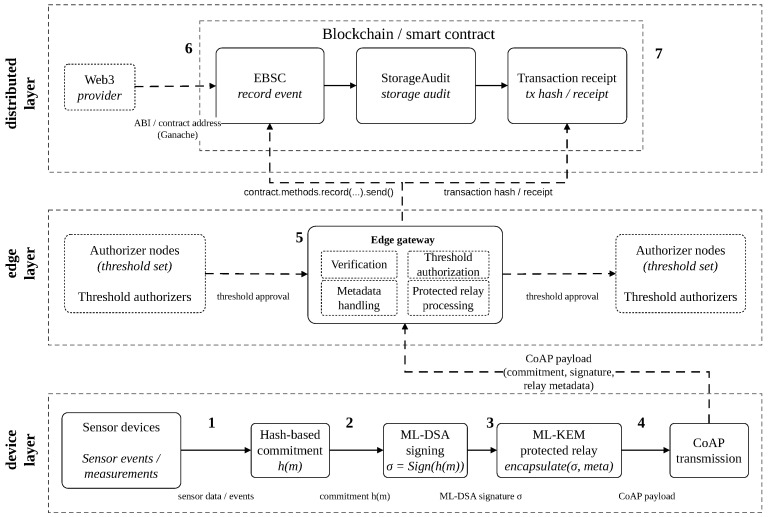
Layered architecture and end-to-end workflow of the proposed CRQC-aware sensor-to-blockchain transaction framework. Numbers 1–7 indicate the workflow order.

This section details the CRQC-aware blockchain transaction framework, which builds upon the system and threat model defined in Section 4. This section formally describes the unique technical components of the proposed framework. The proposed framework offers a transaction security model that evaluates each transaction for $tx_{i}$ using ML-DSA-based signature verification results, QES value, threshold authorization status, relay mode, and key version information. This model combines post-quantum authentication, exposure control, and low-cost on-chain auditing within the same transaction framework. Algorithm 1 summarizes the decision points of the proposed framework. Figure 1 shows the overall layered architecture of the proposed framework.

**Algorithm 1:** CRQC-aware exposure-minimizing transaction control**Input:** $d_{i}$, $pk_{i}$, $sk_{i}$, $kv_{i}$, $policy_{i}$, $ts_{i}$, threshold parameters (t,n), relay public key $pk_{R}$**Output:** EBSC audit event or rejection  1: Compute $h_{i}=H\left(d_{i}\;\|\;ts_{i}\right)$.  2: Compute $pkHash_{i}=H\left(pk_{i}\right)$.  3: Generate $nonce_{i}$.  4: Compute $com_{i}=H(h_{i}\;\|\;pkHash_{i}\;\|\;nonce_{i}\;\|\;kv_{i}\;\|\;policy_{i})$.  5: Collect approval bits $a_{j}$ from authorizing edge nodes $V_{j}$.  6: Compute $Auth_{i}=\left\{\begin{array}{cc} 1, & \mathrm{if}\;\sum_{j=1}^{n}a_{j}\geq t,\\ 0, & \mathrm{otherwise}. \end{array}\right.$  7: If $Auth_{i}=0$, reject $tx_{i}$ or generate a failed-authorization audit event according to $policy_{i}$.  8: Define $relayMode_{i}$ according to the selected relay path.  9: Define $m_{i}=com_{i}\;\|\;kv_{i}\;\|\;Auth_{i}\;\|\;relayMode_{i}\;\|\;ts_{i}$.10: Generate $\sigma_{i}\leftarrow {\mathrm{Sign}}_{\mathrm{ML}-\mathrm{DSA}}\left(sk_{i},m_{i}\right)$.11: Verify $b_{i}\leftarrow {\mathrm{Verify}}_{\mathrm{ML}-\mathrm{DSA}}\left(pk_{i},m_{i},\sigma_{i}\right)$.12: If $b_{i}=0$, reject $tx_{i}$.13: Compute $qes_{i}\leftarrow QES\left(tx_{i}\right)$ using the exposure vector $x_{tx_{i}}$.14: If $qes_{i}$ exceeds the policy-defined critical threshold, reject, delay, rotate the key, or force protected relay according to $policy_{i}$.15: Establish $\left(ct_{R},K_{R}\right)\leftarrow {\mathrm{Encaps}}_{\text{ML-KEM-512}}\left(pk_{R}\right)$.16: Define $M_{i}=com_{i}\;\|\;\sigma_{i}\;\|\;kv_{i}\;\|\;Auth_{i}\;\|\;relayMode_{i}\;\|\;qes_{i}\;\|\;ts_{i}$.17: Define associated data $AD_{i}$ according to the relay session and policy context.18: Encrypt $C_{i}\leftarrow \mathrm{AEAD}.{\mathrm{Enc}}_{K_{R}}\left(M_{i},AD_{i}\right)$.19: Submit protected metadata $\left(ct_{R},C_{i},AD_{i}\right)$ according to the relay policy.20: EBSC emits $\mathrm{EBSC}.\mathrm{emit}(com_{i},kv_{i},Auth_{i},relayMode_{i},qes_{i},ts_{i})$.21: Return the audit event.

### 5.1. Commitment-Based Transaction Representation

The proposed framework represents sensor data through a compact hash-based commitment before the transaction is forwarded to the blockchain layer. First, the sensor data hash is computed as

(7) $$h_{i}=H\left(d_{i}\;\|\;ts_{i}\right).$$

The public-key hash is then generated as

(8) $$pkHash_{i}=H\left(pk_{i}\right).$$

The transaction commitment is defined as

(9) $$com_{i}=H(h_{i}\;\|\;pkHash_{i}\;\|\;nonce_{i}\;\|\;kv_{i}\;\|\;policy_{i}).$$

This commitment binds the sensor data hash, public-key hash, nonce, key version, and policy information into a single transaction representation.

### 5.2. Quantum Exposure Score Formulation

QES is proposed to quantitatively express the transaction-level exposure of a sensor network–blockchain transaction to a CRQC-capable attacker. QES considers factors such as public-key visibility, relay/mempool exposure duration, key reuse, post-quantum migration state, and authorization centralization that occur throughout the transaction lifecycle [29]. The exposure vector for a transaction $tx_{i}$ is defined as follows:

(10) $$x_{tx_{i}}=\left(E_{pk},T_{relay},R_{reuse},A_{sig},M_{pq},S_{central}\right).$$

where $E_{pk}$ represents the public-key exposure level, $T_{relay}$ the relay or mempool exposure duration, $R_{reuse}$ the key reuse, $A_{sig}$ the quantum vulnerability of the signature mechanism, $M_{pq}$ the post-quantum migration readiness, and $S_{central}$ the single-signer or single-gateway dependency.

QES is defined as the weighted sum of normalized exposure components, as follows:

(11) $$\begin{array}{cc} QES(tx_{i})=100\;\times & (w_{1}E_{pk}+w_{2}T_{relay,norm}+w_{3}R_{reuse}\\ \; & +w_{4}A_{sig}+w_{5}\left(1-M_{pq}\right)+w_{6}S_{central}). \end{array}$$

where $w_{1}$, $w_{2}$, $w_{3}$, $w_{4}$, $w_{5}$, and $w_{6}$ are weighting coefficients. The weighting coefficients satisfy the following condition:

(12) $$w_{1}+w_{2}+w_{3}+w_{4}+w_{5}+w_{6}=1.$$

$T_{relay,norm}$ is the normalized version of the relay or mempool visibility time, as follows:

(13) $$T_{relay,norm}=\min\left(\frac{T_{relay}}{T_{max}},1\right).$$

where $T_{max}$ denotes the maximum exposure window permitted by the system policy. As the $T_{relay}$ value approaches or exceeds $T_{max}$, the risk of relay exposure increases.

The default QES weights were selected according to the relative contribution of each component to transaction-level CRQC exposure. Public-key exposure was assigned the highest weight because a visible public key directly increases the attack window of a CRQC-capable adversary. Relay/mempool exposure duration and signature vulnerability were also assigned high weights because they determine whether a transaction can be observed and cryptographically targeted during propagation. Key reuse and post-quantum migration readiness were assigned medium weights, since they affect cumulative exposure and migration robustness. Signer centralization was assigned a lower but non-negligible weight because it mainly captures authorization dependency rather than direct cryptographic breakability. The default QES weights used in this study are summarized in Table 4.

Accordingly, the QES value is computed as

(14) $$\begin{array}{cc} QES(tx_{i})=100\;\times & (0.22E_{pk}+0.20T_{\mathrm{relay},\mathrm{norm}}+0.14R_{\mathrm{reuse}}\\ \; & +0.20A_{\mathrm{sig}}+0.14\left(1-M_{\mathrm{pq}}\right)+0.10S_{\mathrm{central}}). \end{array}$$

All exposure components are normalized to the interval [0,1]. A higher component value indicates higher exposure, except for $M_{pq}$, which represents post-quantum migration readiness. Therefore, $M_{pq}$ is modeled as a mitigating factor through the term $\left(1-M_{pq}\right)$.

The QES weighting scheme follows the relative contribution of each exposure component to the CRQC-aware transaction attack surface considered in this study. Higher weights are assigned to factors that directly affect transaction observability or cryptographic exploitability. Public-key exposure, relay/mempool exposure duration, and signature vulnerability jointly account for 0.62 of the total QES weight because these components represent the primary CRQC-relevant exposure channels in the transaction workflow. Key reuse, post-quantum migration readiness, and signer centralization are also included with lower weights because they influence the persistence of exposure, migration maturity, and authorization robustness. This weighting structure provides a normalized transaction-level exposure index for comparing the evaluated security configurations under the same threat model. The QES value is interpreted between 0 and 100. A low QES value indicates that the transaction carries a lower risk of CRQC-aware exposure. A high QES value indicates increased public key visibility, relay exposure time, key reuse, classical signature dependency, or single-signer dependency. The resulting QES value is mapped to four exposure levels, as summarized in Table 5. This classification enables each transaction to be interpreted not only as a numerical score but also as an audit-level exposure category.

This classification allows the $qes_{i}$ value published by the EBSC to be used not only as a numerical score but also as an audit level. Thus, blockchain event logs represent not only that a transaction occurred but also the CRQC-aware exposure level at which it occurred.

### 5.3. Threshold-Authorized Transaction Control

Threshold-authorized transaction control is a proposed mechanism to reduce dependence on a single gateway or signer. This mechanism requires a transaction to be approved by at least *t* authorizing edge nodes before it is forwarded to the protected relay and EBSC layers.

Each authorizing edge node $V_{j}$ generates an acknowledgment bit for the commitment $com_{i}$ as follows:

(15) $$a_{j}=\left\{\begin{array}{cc} 1, & \mathrm{if}\;V_{j}\;\mathrm{approves}\;com_{i},\\ 0, & \mathrm{otherwise}. \end{array}\right.$$

The threshold authorization result, $Auth_{i}$, is calculated as follows:

(16) $$Auth_{i}=\left\{\begin{array}{cc} 1, & \mathrm{if}\;\sum_{j=1}^{n}a_{j}\geq t,\\ 0, & \mathrm{if}\;\sum_{j=1}^{n}a_{j}<t. \end{array}\right.$$

When $Auth_{i}=1$, the transaction can be forwarded to the protected relay layer. When $Auth_{i}=0$, the transaction is rejected, or, depending on the system policy, the failed authorization event can be logged as a separate audit event. Threshold authorization is reflected in the QES metric via the $S_{central}$ variable. If the transaction is controlled by a single signer or a single gateway, $S_{central}$ is set to 1. If the transaction is approved by multiple authorizers using the t-of-n confirmation rule, $S_{central}$ is set to 0 or a lower intermediate value depending on the system policy. This allows QES to measure not only the choice of cryptographic primitive but also the level of centralization in the authorization process. This structure is particularly important in sensor networks. This is because if the edge gateway alone handles transaction generation, signing, and chain transmission, a central breaking point is created in the system. Threshold authorization reduces the risk of single-signer targeting by spreading this decision point across multiple edge nodes.

In this study, threshold authorization refers to a policy-level t-of-n approval mechanism implemented before the relay forwarding and EBSC event publication stages. Each authorizing edge node evaluates the transaction commitment according to the defined policy and produces an ML-DSA-signed approval message using its own authorization key. The transaction proceeds only when at least t valid approval messages are verified. The resulting authorization state is then bound to the transaction context and included in the ML-DSA-authenticated message processed by the edge gateway. In this structure, ML-DSA provides cryptographic authentication for approval messages and transaction metadata, while threshold authorization provides distributed policy approval and reduces the impact of single-gateway or single-signer dependencies.

### 5.4. Protected Relay and Controlled Metadata Disclosure

The protected relay component is used to reduce the unnecessary visibility of transaction metadata in the public relay or mempool environment. From a CRQC-aware transaction security perspective, relay/mempool visibility is not only a confidentiality issue; it also creates an exposure window during which an attacker can observe the public key, signature, key version information, and transaction timing. In this study, an ML-KEM-512-based session key setup is used for the protected relay. Relay key generation is shown as follows:

(17) $$\left(pk_{R},sk_{R}\right)\leftarrow \mathrm{ML-KEM-}512.\;\mathrm{KeyGen}().$$

The edge gateway encapsulates the session key using the relay public key:

(18) $$\left(ct_{R},K_{R}\right)\leftarrow \mathrm{ML-KEM-}512.\;\mathrm{Encaps}\left(pk_{R}\right).$$

The relay obtains the same session key using its decapsulation key material:

(19) $$K_{R}\leftarrow \mathrm{ML-KEM-}512.\;\mathrm{Decaps}\left(ct_{R},sk_{R}\right).$$

The transaction metadata $M_{i}$ is defined as follows:

(20) $$M_{i}=com_{i}\;\|\;\sigma_{i}\;\|\;kv_{i}\;\|\;Auth_{i}\;\|\;relayMode_{i}\;\|\;qes_{i}\;\|\;ts_{i}.$$

This metadata is protected using authenticated encryption with associated data (AEAD), with the session key $K_{R}$:

(21) $$C_{i}={\mathrm{AEAD.}\;\mathrm{Enc}}_{K_{R}}\left(M_{i},AD_{i}\right).$$

where $AD_{i}$ is the unencrypted but verified additional data (associated data). This field can contain values such as a policy, a gateway identifier, a relay session identifier, or a timestamp. The protected relay receives the $C_{i}$ value, performs the verification or routing operation according to the policy, and ensures that only the necessary audit metadata reaches the EBSC layer. This design applies the controlled metadata disclosure principle. This means that not every transaction field is published at the same level of visibility. The use of a protected relay has two effects on QES. Firstly, when $relayMode_{i}=$ protected, the $T_{r}elay_{n}orm$ value decreases. Secondly, because the process metadata is carried over a shorter or more restricted relay/mempool, the $E_{p}k$ and $T_{r}elay$ components can be assigned lower values. Conversely, when $relayMode_{i}=$ open, the $T_{r}elay_{n}orm$ and $E_{p}k$ values are set higher per the system policy.

### 5.5. ML-DSA-Based Transaction Authentication

ML-DSA-based transaction authentication enables post-quantum-secure verification of the transaction commitment and its associated metadata.

The message $m_{i}$ to be signed is defined as follows:

(22) $$m_{i}=com_{i}\;\|\;kv_{i}\;\|\;Auth_{i}\;\|\;relayMode_{i}\;\|\;ts_{i}.$$

The ML-DSA signature is generated as follows:

(23) $$\sigma_{i}\leftarrow {\mathrm{Sign}}_{\mathrm{ML}-\mathrm{DSA}}\left(sk_{i},m_{i}\right).$$

Verification is performed as follows:

(24) $$b_{i}\leftarrow {\mathrm{Verify}}_{\mathrm{ML}-\mathrm{DSA}}\left(pk_{i},m_{i},\sigma_{i}\right).$$

If $b_{i}=1$, the transaction is considered valid for authentication. If $b_{i}=0$, the transaction is rejected. In this design, the signature is not solely dependent on the data commitment; the signature is created along with the transaction context. Therefore, the reuse of the same $com_{i}$ value across different key versions, relay modes, or threshold authorization results is prevented. This context-bound authentication approach aims to reduce the risks of transaction replay, context substitution, and metadata manipulation. The ML-DSA signature is used in the validation layer. However, it is not stored persistently within EBSC. This is because post-quantum signature outputs are larger than classical signatures, increasing gas and storage overhead when written directly to the chain. Therefore, the proposed framework uses ML-DSA for transaction authentication while publishing only verifiable, compact audit metadata on EBSC.

### 5.6. EBSC-Based Low-Cost Audit

EBSC is the low-cost blockchain audit layer of the proposed framework. The main purpose of EBSC is to ensure the auditability of sensor data commitments on the chain without storing raw data, full public keys, or large ML-DSA signature outputs in contract storage [30]. The EBSC event structure is defined as follows:

(25) $$\mathrm{EBSC.}\;\mathrm{emit}(com_{i},kv_{i},Auth_{i},relayMode_{i},qes_{i},ts_{i}).$$

These event fields carry the following functions: $com_{i}$ is the transaction commitment value associated with the sensor data; $kv_{i}$ represents the key version used in the transaction; $Auth_{i}$ shows the threshold authorization result; $relayMode_{i}$ indicates whether the transaction is carried over a protected or open relay; $qes_{i}$ denotes the transaction-level quantum exposure; and $ts_{i}$ represents the transaction timestamp. In a storage-based smart contract approach, data or large amounts of metadata are written to the contract state. In this case, each record creates a persistent contract storage cost. The EBSC approach, however, provides the control record via the event log. Therefore, EBSC aims to reduce on-chain storage overhead and gas costs compared to the storage-based approach.

### 5.7. Security-Oriented Properties

The proposed framework offers security features that simultaneously support cryptographic verification, exposure control, authorization distribution, and low-cost on-chain audit objectives within the sensor network–blockchain transaction flow.

**Property** **1** (Commitment-based data abstraction). *Sensor data are represented through the hash-based commitment construction defined in Equations (7)–(9). This construction binds the sensor data hash, public-key hash, nonce, key version, and policy information into a compact transaction representation. As a result, raw sensor data are not exposed at the blockchain layer, while integrity verification remains possible through the commitment value.*

**Property** **2** (Context-bound ML-DSA authentication). *The ML-DSA signature is generated over a context-bound transaction message. The message to be signed is defined in Equation (22). This message binds the transaction commitment, key version, threshold authorization result, relay mode, and timestamp into a single authentication context. The signature is generated as shown in Equation (23) and verified according to Equation (24). Therefore, signature verification is not performed only over an isolated transaction value, but over a transaction representation that includes data commitment, key lifecycle information, authorization status, relay preference, and temporal context.*

**Property** **3** (Protected relay visibility reduction). *At the protected relay layer, the session key $K_{R}$ established using ML-KEM-512 restricts the readable exposure of transaction metadata in open relay or public mempool environments. The transaction metadata is defined using Equation (20) and encrypted via Equation (21). An attacker observing the relay or mempool cannot directly access fields such as the transaction commitment, signature, key version, authorization state, relay mode, and QES value. This property reduces the impact of relay exposure and on-spend exposure windows.*

**Property** **4** (Threshold-based authorization distribution). *The threshold authorization mechanism distributes the decision to approve a transaction across multiple authorizing edge nodes. According to the threshold rule defined in Equations (15) and (16), a transaction is forwarded only if at least (t) out of (n) authorizing nodes approve the commitment. This threshold rule reduces the concentration of control on a single gateway or signer during transaction generation and on-chain transfers. By distributing the authorization decision, the proposed framework reduces single-signer dependence and adds an additional control layer against centralized signer targeting within the CRQC-aware transaction risk model.*

**Property** **5** (Metadata-minimized blockchain audit). *The EBSC audit event defined in Equation (25) emits only compact audit metadata. Since raw sensor data, large transaction metadata, and ML-DSA signature outputs are not stored in contract storage, the on-chain storage overhead is reduced. Thus, the EBSC structure lowers gas cost and on-chain overhead compared with storage-based smart contract designs.*

**Property** **6** (Quantified CRQC-aware transaction exposure). *The QES formulation defined in Equations (10)–(13) aggregates public-key exposure, relay/mempool visibility, key reuse, signature vulnerability, post-quantum migration readiness, and signer centralization factors into a measurable transaction-level risk score. Each transaction is evaluated according to its QES value together with the ML-DSA validation result. Adding the QES value to the EBSC event log provides the on-chain audit layer with an exposure-aware monitoring capability under the CRQC-aware transaction risk model.*

## 6. Experimental Results and Discussion

This section presents an experimental evaluation of the proposed CRQC-aware sensor-to-blockchain transaction framework. The evaluation was performed on real DPV/ECS electrochemical toxicity sensor data. The experimental analysis addresses cryptographic operation time, communication overhead, off-chain processing latency, blockchain gas cost, QES-based exposure reduction, and metric-level comparison with related studies. The experimental evaluation was designed to measure the end-to-end sensor-to-blockchain transaction processing capabilities and CRQC-aware exposure minimization effect of the proposed framework on real DPV/ECS sensor records. For this purpose, the transaction times of the ML-KEM-512, ML-DSA, threshold-based transaction authorization, ML-KEM-512 protected relay, and hash-based commitment components running on the edge gateway were evaluated separately. Furthermore, the payload and chunk costs of CoAP-based sensor transfer, the gas advantage of the EBSC-based on-chain mechanism compared to the storage-based smart contract approach, and the exposure separation capability of the QES metric in different transaction security scenarios were evaluated under scalability scenarios. The evaluation in this section is structured around three measurable output groups. The first output group comprises processing-time metrics, demonstrating post-quantum transaction feasibility. The second output group comprises gas consumption and communication cost metrics, which indicate blockchain overhead. The third output group comprises scenario-based QES values that demonstrate CRQC-aware exposure minimization.

### 6.1. Experimental Setup and Dataset

The experimental evaluation was performed on a local computer with an Intel i7 processor and 32 GB of RAM. The edge gateway and sensor client components were developed using Node.js. CoAP was used for sensor data transfer. The blockchain layer was run on a local Ethereum-compatible Ganache network. EBSC and StorageAudit smart contracts were deployed to the Ganache network via Remix IDE. Interaction with the blockchain was provided through Web3.js.

For post-quantum cryptographic operations, PQClean-based ML-KEM-512 and ML-DSA components were compiled as WebAssembly and integrated into the Node.js-based edge gateway layer. PQClean is a framework that aims to provide clean, portable, and dependency-reduced C implementations of algorithms submitted to the NIST post-quantum standardization process using a common API [31]. PQClean’s structure, which reduces external dependencies, facilitates the integration of different post-quantum primitives into existing applications. Furthermore, compliance with the C standard, consistent compilation rules, standardization in integer representation, and checklists aimed at improving source code quality make PQClean suitable for experimental post-quantum prototyping. In this study, PQClean-based ML-KEM-512 and ML-DSA implementations were compiled into WASM modules and integrated into a sensor-to-blockchain transaction pipeline.

The sensor data used consists of real DPV/ECS electrochemical recordings obtained under doxorubicin exposure. The data were obtained from PLL@SPCE-based cell culture platforms. The dataset includes dose, exposure time, electrode ID, and measured current value. These recordings were converted into sensor-to-blockchain transaction objects. Since the original biological sensor record count was lower than the number of transactions used in the scalability test, the actual measurements were replayed with unique sample identifiers, timestamps, key-version metadata, and transaction-level metadata. The experimental overhead was based on the actual DPV/ECS sensor measurements. The throughput was expanded to evaluate the system’s scalability.

Experiments were conducted at scales of 100, 500, 1000, 2500, 5000, and 10,000 transactions. Each scale was repeated three times in the main transaction-processing benchmark, and a total of 57,300 sensor-to-blockchain transactions were processed. For each transaction, CoAP reception, sensor hashing, commitment generation, ML-DSA signing and verification, threshold-based authorization, ML-KEM-512 protected relay processing, AEAD encryption/decryption, and QES computation were performed. For the blockchain-side audit evaluation, a full on-chain experiment was conducted in which every sensor transaction was recorded on-chain using both EBSC and StorageAudit. At the largest scale, 10,000 sensor transactions generated 20,000 blockchain transactions in total. This total consisted of 10,000 EBSC audit records and 10,000 StorageAudit records. In addition, a latency/jitter-aware full on-chain experiment was conducted at the 10,000-transaction scale using controlled communication delay profiles.

### 6.2. Cryptographic Performance

Table 6 shows the average cryptographic transaction times for different transaction scales. The results show that the post-quantum operations run with low latency on the edge gateway. The ML-KEM-512 encapsulation time remained in the range of 0.124–0.140 ms at all scales. The ML-KEM-512 decapsulation time was measured to be 0.149–0.163 ms. These values show that the post-quantum key establishment used for the protected relay adds low overhead to the overall transaction pipeline.

The ML-DSA signing time ranged from 0.245 to 0.264 ms, and the ML-DSA verification time ranged from 0.070 to 0.071 ms. This result shows that using ML-DSA for transaction authentication is feasible at the edge gateway layer. Although the ML-DSA signature size is larger than that of classical signature schemes, the proposed system does not write large signature outputs to on-chain storage.

The threshold-based authorization process consists of two main operations. First, authorizer nodes evaluate the transaction commitment according to the defined policy and return ML-DSA-signed approval messages generated with their own authorization keys. Second, the edge gateway verifies these approval messages and counts the valid approvals until the t-of-n policy threshold is reached. The threshold collection time was approximately 0.737–0.755 ms, and the threshold verification time was approximately 0.213–0.217 ms. These results show that the t-of-n signed-approval mechanism reduces single-signer dependency while adding a manageable transaction cost of approximately 1 ms.

The AEAD encryption and decryption operations were measured to be approximately 0.014–0.030 ms and 0.007–0.011 ms, respectively. The commitment generation time was approximately 0.002–0.003 ms across all scales. This value indicates that the hash-based commitment structure adds a negligible overhead to the pipeline.

**Figure 2:**
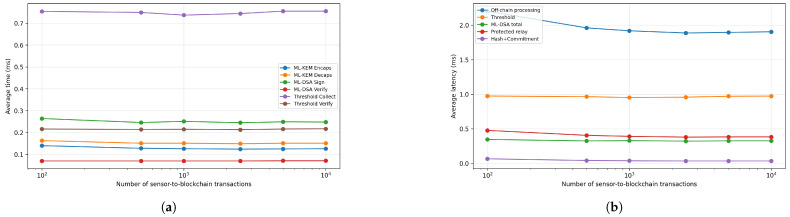
Performance and scalability overview of the proposed framework. (**a**) Cryptographic operation times. (**b**) Off-chain processing latency by scale.

Figure 2a shows cryptographic operation times by scale. No significant increase was observed in the ML-KEM, ML-DSA, threshold authorization, and commitment generation times as the scale increased from 100 to 10,000 transactions. This indicates that the proposed edge-side post-quantum transaction-processing layer remains stable as transaction throughput increases.

### 6.3. Latency and Scalability Analysis

Table 7 shows the latency breakdown of the proposed framework. In this study, the primary metric for scalability is off-chain processing latency. Off-chain processing latency is the sum of the following components: CoAP reassembly, hash and commitment generation, threshold authorization, ML-DSA operation, and protected relay processing. This metric directly reflects the transaction cost of the sensor-to-blockchain transaction pipeline before the optional on-chain audit step.

According to the results, off-chain processing latency was measured as 2.202 ms for 100 transactions, 1.960 ms for 500 transactions, 1.918 ms for 1000 transactions, 1.887 ms for 2500 transactions, 1.895 ms for 5000 transactions, and 1.904 ms for 10,000 transactions. The fact that latency did not increase as scale increased indicates that the framework operates stably on the edge-gateway side. Higher average values observed at smaller scales can be explained by runtime warm-up, initial costs, and the higher impact of blockchain records on the average.

Figure 2b shows the off-chain processing latency trend. Threshold-based authorization is the largest off-chain latency component, followed by protected relay processing and ML-DSA processing. Hash and commitment generation have a very low latency across all tested scales. These results show that the edge-side transaction-processing pipeline remains stable up to the 10,000-transaction scale. Blockchain-side full audit behavior, including on-chain recording latency and gas cost, is evaluated separately in Section 6.4 using full on-chain audit experiments.

### 6.4. Communication and Blockchain Overhead Analysis

**Figure 3:**
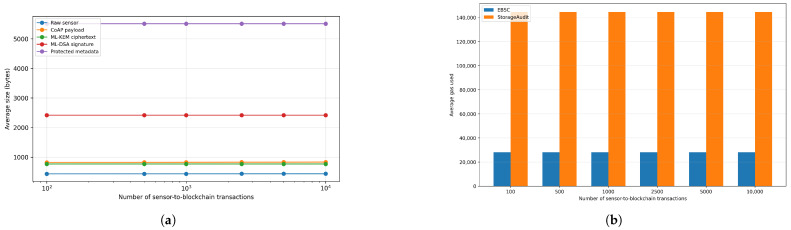
Communication and blockchain overhead analysis. (**a**) Communication overhead by scale. (**b**) EBSC vs. StorageAudit gas comparison.

The communication overhead results indicate that the proposed workflow introduces a stable and predictable payload size across different transaction scales. As shown in Figure 3a, the raw sensor payload remains approximately 435–438 bytes, while the CoAP payload remains around 823–833 bytes from 100 to 10,000 transactions. This shows that the CoAP-based transmission does not create a scale-dependent communication burden for real DPV/ECS sensor records. The larger protected metadata size is expected because each transaction carries not only the measurement value but also commitment, post-quantum authentication, protected relay, and audit-related metadata. However, ML-KEM ciphertext, ML-DSA signature, and protected metadata sizes remain constant across all scales. Therefore, the communication cost is mainly determined by the selected security primitives and transaction format, not by the number of processed transactions. This makes the proposed workflow suitable for predictable large-scale sensor-to-blockchain transaction processing.

Figure 3b shows a comparison of gas costs between the proposed EBSC audit layer and the storage-based StorageAudit contract. Both contracts were tested using the same audit metadata fields. EBSC publishes compact audit metadata as an event, while StorageAudit stores the same metadata fields on the contract storage. According to the experimental results, EBSC consumed an average of approximately 28,036 gas, while StorageAudit consumed an average of approximately 144,464 gas. This result shows that EBSC provides an approximately 80.59% gas reduction compared to the storage-based audit approach. This rate remained almost constant across all scales. The main reason is that EBSC does not perform persistent storage writes and only generates event logs.

This result demonstrates that the proposed EBSC layer effectively reduces blockchain overhead. In post-quantum transaction frameworks, writing large cryptographic outputs such as ML-DSA signatures and ML-KEM ciphertexts directly to on-chain storage would be costly. In the proposed system, raw sensor data, large post-quantum signature outputs, and protected relay ciphertexts are not written to the chain. Commitment, key version, threshold status, relay mode, QES level, and timestamp are published as compact metadata events. This significantly reduces on-chain costs while maintaining auditability. A full on-chain audit experiment was conducted to evaluate the scalability of blockchain-side audits. In this experiment, each sensor transaction level, ranging from 100 to 10,000, was recorded on-chain using both EBSC and StorageAudit. Therefore, a total of 20,000 blockchain transactions were generated at the largest full-on-chain setting. This contained a total of 10,000 EBSC audit records and 10,000 StorageAudit records.

As shown in Table 8, EBSC maintained a stable average gas cost of approximately 28,036 gas across all tested scales. StorageAudit required approximately 144,464 gas. This indicates an approximate 80.59% gas reduction across all full on-chain experiments. The results confirm that the event-based audit design provides a consistent blockchain cost advantage per record when all transactions are recorded on-chain. In the local Ganache environment, the average chain latency remained within a narrow range, from 14.631 ms to 18.510 ms.

To evaluate the behavior of the proposed workflow under non-zero communication delay, a full on-chain latency- and jitter-aware audit experiment was conducted at a scale of 10,000 sensor transactions. In this experiment, each sensor transaction was recorded on-chain using both EBSC and StorageAudit. Each latency/jitter profile generated 20,000 blockchain transactions. This total included 10,000 EBSC audit records and 10,000 StorageAudit records. Two latency/jitter profiles were evaluated. The first profile used a 10 ms base delay and 5 ms jitter. This setting produced an average simulated delay of 12,504 ms. The second profile used a 50 ms base delay and 20 ms jitter. This setting produced an average simulated delay of 59,935 ms. As seen in Table 9, the total latency increased in line with the injected network delay. Under the lat10-jit5 profile, the average processing latency was 27.892 ms, and the average total latency, including network delay, was 40.396 ms. Under the lat50-jit20 profile, the average processing latency was 39.490 ms, and the average total latency, including network delay, was 99.424 ms. The observed latency increase closely followed the average simulated delay in each profile. These results demonstrate that the proposed workflow remains operational under controlled latency/jitter conditions, and that latency values change as expected when network delay increases.

### 6.5. QES-Based Exposure Analysis

In the classical open transaction scenario, public-key visibility, relay/mempool exposure, key reuse, classical signature vulnerability, lack of post-quantum migration readiness, and single-signer dependency are at their highest levels. Therefore, the QES value was calculated as 100 and classified as a critical exposure. Table 10 shows the QES values for four different transaction security scenarios. In the ML-DSA-only scenario, the signature mechanism is post-quantized. However, relay visibility, key reuse, and single-signer dependency factors persist. Therefore, although the QES value drops to 64.4, it remains at the high-exposure level. This result shows that the signature-algorithm replacement approach alone is insufficient to fully reduce the risk of CRQC-aware transaction exposure. In the ML-DSA + protected relay scenario, relay visibility is reduced in addition to the post-quantum signature mechanism. In this case, the QES value drops to 32.4, placing it at the moderate exposure level. In the full framework scenario, ML-DSA, ML-KEM-512 protected relay, threshold-based authorization, key lifecycle metadata, and EBSC are used together. In this case, the QES value is reduced to 4 and classified as low exposure.

**Figure 4:**
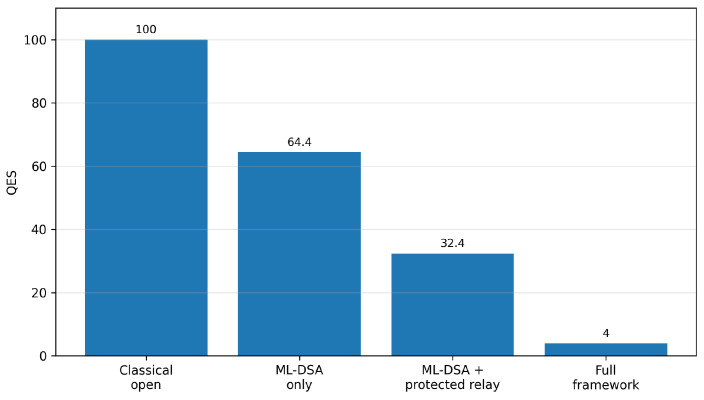
QES-based exposure reduction across transaction security scenarios.

Figure 4 shows the percentage reduction in the QES value across the evaluated transaction security scenarios. Compared with the classical open transaction scenario, the ML-DSA-only configuration reduces the exposure score by 35.6%. The ML-DSA with a protected relay configuration reduces the exposure score by 67.6%. The full framework reduces the exposure score by 96%. These results show that the highest reduction is achieved when post-quantum authentication, protected relay communication, threshold authorization, key lifecycle metadata, and EBSC-based audit are used together within the same transaction workflow. The QES-based evaluation reports the exposure reduction achieved by each transaction security configuration as percentage values. The QES reduction from 100 to 4 follows from the fixed QES formulation and the normalized scenario inputs. In the classical open transaction scenario, all exposure components are active, which yields a QES value of 100. In the full-framework scenario, public-key exposure, key reuse, signature vulnerability, missing post-quantum migration readiness, and signer centralization are minimized in the QES model. The residual score is produced by the protected relay exposure term. With a baseline relay/mempool exposure weight of 0.20 and a normalized protected relay exposure value of 0.2, the full-framework QES value is calculated as 100 × (0.20 × 0.2) = 4. This value corresponds to the remaining workflow-level exposure captured by the QES model. A sensitivity analysis was conducted to examine the robustness of the QES-based comparison under alternative weight settings. Four weight settings were evaluated. The baseline setting used the default QES weights. The critical +10% and critical −10% settings increased or decreased the three direct CRQC-related factors, namely public-key exposure, relay/mempool exposure duration, and signature vulnerability, by 10% and then renormalized all weights. The balanced setting assigned equal weight to all six QES components. Table 11 presents the QES sensitivity results under these alternative weight settings.

The results show that the relative ordering of the evaluated scenarios remains unchanged under all tested weight settings. The classical open transaction scenario has the highest QES value, followed by the ML-DSA-only configuration and the ML-DSA with the protected relay configuration. The full framework has the lowest QES value across all settings, ranging from 3.33 to 4.14. These results indicate that the QES-based comparison remains stable under the evaluated weight perturbations.

### 6.6. Same-Environment Ablation and Baseline Comparison

To evaluate the contribution of each framework component individually, an ablation experiment was performed in the same experimental environment at a scale of 1000 sensor transactions. All configurations were run using the same Node.js edge gateway, PQClean/WASM cryptographic execution layer, CoAP sensor pipeline, Ganache-based Ethereum-compatible blockchain environment, and actual DPV/ECS sensor dataset. This setup enables a controlled comparison of signature mode, relay protection, threshold authorization, and audit contract design under identical prototype conditions. The 1000-operation scale was chosen as a fixed and repeatable baseline to compare module-level impacts without repeating the entire scalability analysis.

Five different configurations were evaluated. The classical open baseline uses ECDSA-based edge authentication, open relay transmission, and threshold authorization at the edge-authentication layer. The ML-DSA-only configuration replaces the classical signature layer with ML-DSA, leaves the relay open, and disables threshold authorization. The protected relay configuration adds ML-KEM-512 and AEAD-based relay protection without threshold authorization. The threshold open relay configuration enables threshold authorization while the relay remains open. The full framework integrates ML-DSA, protected relay processing, threshold authorization, and event-based audit recording components. In all configurations, EBSC and StorageAudit were run in the same Ganache environment.

The ablation results in Table 12 show that each module contributes differently to the transaction security profile. Replacing the classical signature layer with ML-DSA reduces the CRQC-related signature vulnerability value and lowers the QES value from 100.0 to 64.4. Adding a protected relay further reduces the relay-level exposure value, lowering the QES value to 32.4. Enabling threshold authorization over an open relay reduces single-signer dependency and yields a QES value of 40.4. This intermediate result separates the effect of threshold authorization from the effect of relay protection. While threshold authorization improves the signer-centralization component, open relay still retains the effect of relay/mempool exposure. The full framework achieves a minimum QES value of 4.0 by using ML-DSA, protected relay processing, and threshold authorization components, with an average total latency of 17.164 ms.

The ablation results from the same experimental environment show that the proposed framework primarily reduces CRQC-aware transaction exposure through the combined use of ML-DSA authentication, protected relay processing, and threshold authorization components. The results also demonstrate that these security gains are achieved in a controlled prototype environment along with stable latency, communication overhead, and audit-layer behavior.

### 6.7. Metric-Level Quantitative Comparison with Related Studies

The experimental results of the proposed framework were evaluated using a metric-level quantitative comparison approach with related post-quantum blockchain, IoT, edge-assisted verification, and smart contract audit studies. Directly comparing absolute time or throughput in such studies is not always reliable. This is primarily because existing studies use different hardware platforms, blockchain environments, network conditions, workload structures, and PQC primitives. Therefore, the comparison was structured through the quantitative metric categories of the relevant studies. Measurement dimensions such as PQ signature cost, edge verification latency, storage overhead, throughput, smart contract gas consumption, and communication overhead were considered separately, and how the proposed framework encompasses these metrics within a unified sensor-to-blockchain transaction workflow was demonstrated.

Table 13 compares the proposed framework with related studies at the metric level. The comparison summarizes the platform or context used for each study, the core security focus, quantitative metrics, and their relationship to the proposed framework. This table shows that the proposed study unifies the individual performance evaluations in the existing literature under a more holistic sensor-to-blockchain transaction workflow. Existing studies generally focus on a specific performance axis. PQC-based IoT studies mostly measure key generation, signing, and verification times. Edge-assisted blockchain studies highlight the advantages of verification latency and edge offloading. Blockchain-based IoT security studies evaluate system metrics such as latency, throughput, and storage overhead. Smart contract-focused studies, on the other hand, use gas consumption, execution time, and storage cost as key comparison points. The proposed framework evaluates real DPV/ECS sensor data within a unified transaction pipeline. Experimental results show that ML-KEM-512 and ML-DSA operations can be executed with low latency on the edge gateway, while threshold-based authorization and protected relay layers introduce manageable overhead. In addition, CoAP transmission works with a low chunk count for actual sensor payloads, EBSC provides an approximately 80.59% gas reduction compared to the storage-based audit approach, and QES distinguishes different transaction security scenarios. Therefore, the quantitative contribution of the proposed framework is evident in the combined measurement of PQC execution, communication overhead, off-chain latency, blockchain audit cost, and CRQC-aware exposure scoring metrics.

### 6.8. Discussion

The experimental results demonstrate that the proposed CRQC-aware sensor-to-blockchain framework is practical and scalable under the evaluated conditions. The off-chain processing latency remained at approximately 1.9 ms even at a scale of 10,000 transactions. This result shows that the edge gateway can support post-quantum transaction processing with low overhead. The stability of the cryptographic transaction times with increasing scale demonstrates that ML-KEM-512 and ML-DSA can be effectively integrated into a Node.js/WASM-based edge environment. The results also show that the threshold-based transaction authorization component constitutes the most significant off-chain transaction cost. This is expected, as multiple authorizer approvals are generated and validated. However, this cost reduces the single-signer dependency. Single-signer dependency is a crucial component within the CRQC-aware attack surface. Protected relay operation also introduces additional latency, but it remains below 0.5 ms on average and directly reduces relay/mempool visibility. The latency/jitter-aware full on-chain experiment also showed that end-to-end latency follows the injected communication delay under controlled delay profiles.

Gas analysis demonstrates the importance of the EBSC design. Compared to a storage-based audit contract, EBSC provides an approximately 80.59% gas reduction. This result is particularly important for post-quantum transaction frameworks, as post-quantum signature and ciphertext outputs are larger than their classical counterparts. Writing this data directly to the contract storage area would increase blockchain overhead. The proposed EBSC design reduces this cost by publishing only compact, exposure-aware metadata. QES analysis also supports this study’s main claim. The full on-chain audit results further show that the per-record audit-cost behavior remains stable when all sensor transactions are recorded on-chain. Although the ML-DSA-only scenario reduces signature-level vulnerability, it does not sufficiently reduce transaction-level exposure. The reduction in the QES value from 100 to 4 in the full framework scenario indicates that the CRQC-aware transaction security approach should be addressed at the workflow level. Therefore, the proposed framework offers not only post-quantum signature replacement but also an exposure-minimizing transaction workflow design. From a quantum-security perspective, post-quantum blockchain and quantum blockchain represent related but different design directions. Post-quantum blockchain preserves the conventional blockchain workflow and strengthens it with quantum-resistant cryptographic primitives, whereas quantum blockchain or QKD-assisted blockchain may involve quantum communication, quantum key distribution, or quantum network components. Studies on quantum key distribution systems show that quantum-security evaluation should also consider implementation-level vulnerabilities, including synchronization attacks [33], calibration attacks [34], and metadata-handling processes. These studies indicate that even when a quantum protocol is theoretically secure, relay, synchronization, calibration, and auxiliary communication processes may create additional attack surfaces [33,34]. This perspective supports the workflow-level design adopted in this study, where post-quantum authentication is combined with protected relay communication, metadata minimization, threshold authorization, key lifecycle metadata, and QES-based exposure assessment to reduce CRQC-induced transaction-level exposure in the sensor-to-blockchain process.This study has some limitations. The experimental evaluation was conducted in a controlled prototype environment using a Node.js/WASM-based edge gateway and a Ganache-based Ethereum-compatible blockchain. Therefore, the reported latency values should be interpreted as controlled prototype measurements rather than production network measurements. The full on-chain audit and latency/jitter-aware experiments strengthen the blockchain-side audit evaluation by recording all sensor transactions on-chain and by introducing controlled communication delay profiles. However, the latency/jitter-aware experiment remains an emulation-based evaluation. It does not fully model constrained physical sensor nodes, low-power edge hardware, wireless packet loss, geographically distributed relays, multiple independent edge gateways, or private/consortium blockchain validators. In addition, the DPV/ECS dataset was replayed using unique transaction identifiers and timestamps to evaluate transaction volumes beyond the original number of biological measurements.

## 7. Security Analysis

This section presents a structured security analysis for the proposed CRQC-aware sensor-to-blockchain transaction framework. The analysis examines how standard post-quantum cryptographic building blocks are positioned within the proposed transaction workflow and the impact of this positioning on public-key exposure, relay/mempool visibility, key reuse, single-signer dependency, replay risk, and on-chain audit overhead. ML-KEM and ML-DSA are used as NIST-standardized post-quantum primitives [8,9]. The SHA-3 family is considered the standard hash function family for hash, digest, and commitment generation [28]. This analysis systematically examines which risk each security component in the proposed framework reduces within the transaction workflow. Hash-based commitment is used to reduce the on-chain vulnerability of sensor data. ML-DSA strengthens the transaction authentication process with post-quantum security assumptions. ML-KEM-512 and AEAD limit the visibility of transaction metadata in the open relay/mempool environment at the protected relay layer. Threshold-based authorization reduces single-signer dependency by distributing transaction approval decisions across multiple authorizers. EBSC provides auditability by publishing compact, exposure-aware metadata and reducing storage-heavy blockchain audit costs. QES quantifies CRQC-aware transaction exposure by considering public key exposure, relay visibility, key reuse, signature vulnerability, migration readiness, and signer centralization. The security motivation of the proposed framework is based on the CRQC-capable adversary model.

### 7.1. Security and Reliability Evaluation Criteria

In this study, security and reliability were evaluated based on measurable and verifiable criteria defined at the transaction workflow level. Security evaluation was conducted using criteria such as raw sensor data exposure reduction, context-bound post-quantum authentication, transaction integrity, protected relay-based metadata visibility reduction, threshold-based reduction in single-signer dependency, EBSC-based auditability, and quantified CRQC-aware exposure using QES.

Reliability evaluation was addressed by examining the stable operation of the experimental transaction pipeline at different transaction scales. In this context, reliability was evaluated by completing the following steps within the experimental pipeline: CoAP-based sensor data reception, commitment generation, ML-DSA signing and verification, threshold authorization, ML-KEM-based protected relay, AEAD-based metadata protection, QES computation, and EBSC event emission. Furthermore, the fact that the total off-chain processing latency remained within a limited range across the evaluated scales, that the CoAP payload size and cryptographic metadata sizes did not vary with scale, and that the EBSC gas cost remained consistent were used as reliability indicators. This study does not specifically model blockchain consensus reliability, sensor hardware failures, or lossy network conditions.

### 7.2. Security Objectives

The proposed framework is designed to satisfy the following security objectives.

1. The first objective is to reduce on-chain data exposure. Raw sensor data is not written directly to blockchain storage. Instead, sensor data is represented by hash and commitment values. This structure reduces exposure of on-chain raw data while maintaining auditability.
2. The second objective is to strengthen the transaction authentication process against CRQC-capable adversaries. Elliptic-curve-based signature mechanisms, such as Classical ECDSA and Schnorr, can be vulnerable to CRQC [5,6]. Therefore, the proposed framework uses ML-DSA-based post-quantum signing for transaction authentication [26].
3. The third objective is to reduce relay/mempool visibility. Explicitly transporting transaction metadata in a public relay or mempool environment can increase the risk of CRQC-aware public-key exposure. Therefore, session keys are established with ML-KEM-512 in the protected relay layer, and transaction metadata is protected with AEAD [8,25].
4. The fourth objective is to reduce single-signer dependency. Transaction approval based on a single gateway or single signer is a weak point both operationally and in terms of security. The proposed threshold-based authorization structure reduces this dependency by distributing the transaction approval decision across multiple authorizers.
5. The fifth objective is to keep blockchain overhead low while ensuring auditability. EBSC publishes compact exposure-aware metadata instead of large post-quantum signature or ciphertext outputs. Thus, gas costs are reduced compared to storage-based audit approaches while maintaining a verifiable audit record.
6. The sixth objective is to make CRQC-aware exposure measurable. QES enables quantitative monitoring of transaction-level risk by evaluating public-key exposure, relay visibility, key reuse, signature vulnerability, migration readiness, and signer centralization.

### 7.3. Adversary Capabilities

The security analysis considers the following attacker capabilities:

1. **CRQC-capable attacker:** This attacker is one who can target classical public-key cryptography structures at sufficient scale and with fault-tolerant quantum computing capabilities. In this model, classical public-key structures such as ECDSA, Schnorr, RSA, and BLS are at risk [5,6]. The proposed framework reduces the risk from classical public-key exposure by using ML-DSA for transaction authentication and ML-KEM for protected relay [8,9].
2. **Relay/mempool observer:** This attacker is a passive or semi-active observer who attempts to monitor transaction metadata, public keys, key versions, timestamps, and transaction propagation information. Even without obtaining raw sensor data, they can create an exposure window by exploiting metadata visibility within the transaction flow. The protected relay layer is used to mitigate this visibility.
3. **Compromised single-gateway attacker:** This attacker targets single-gateway or single-signer dependencies. If transaction approval is concentrated on a single gateway, compromising that gateway can weaken the transaction authorization process. Threshold-based authorization reduces this risk by requiring at least t valid approvals.
4. **Replay attacker:** This attacker attempts to reuse previously generated transaction metadata, signatures, or commitment values. In the proposed framework, context binding is provided to defend against replay attempts by including timestamps, nonces, key versions, and policy metadata in the transaction message.
5. **On-chain storage observer:** This attacker refers to an observer who persistently analyzes data published on the blockchain. Due to the blockchain’s append-only and public audit features, on-chain published data can remain visible for a long time. Therefore, the proposed framework does not directly write raw sensor data, ML-DSA signature outputs, or ML-KEM ciphertext values to on-chain storage.

### 7.4. Claim-Based Security Analysis

In this section, the security impact of the proposed framework is examined using a claim-based analysis approach. Each claim indicates the transaction-level risk reduction provided by a specific mechanism within the framework.

**Claim 1. Commitment-based abstraction reduces on-chain raw sensor data exposure.**

Sensor data ($d_{i}$) is not directly represented at the on-chain record layer. The edge gateway first generates the ($h_{i}=H\left(d_{i}\;\|\;ts_{i}\right)$) value, then calculates the ($com_{i}=H(h_{i}\;\|\;pkHash_{i}\;\|\;nonce_{i}\;\|\;kv_{i}\;\|\;policy_{i})$) commitment value. The blockchain audit layer receives commitment-based metadata instead of raw data.

**Justification:** This structure prevents the raw sensor data value from being permanently visible in the on-chain storage. Assuming the collision resistance of the SHA-3-based hash function, it is not practically expected that an attacker would generate the same commitment value from different sensor data ($d_{i}^{\prime }$) [28]. Thus, on-chain data exposure is reduced while data integrity and auditability are preserved.
**Claim 2. ML-DSA-based authentication provides post-quantum transaction authenticity under the assumed unforgeability of ML-DSA.**

In the proposed framework, the signed message does not consist solely of the commitment value; it is constructed as a transaction message. Thus, the signature is linked to the commitment, key version, authorization status, relay mode, and timestamp.

**Justification:** ML-DSA is a module-lattice-based digital signature standard, standardized by NIST [9]. Dilithium’s basic structure is based on module-lattices and the Fiat–Shamir-with-aborts approach [26]. An attacker who wants to create a valid forged transaction needs to generate a valid ML-DSA signature for a new, unapproved transaction context. This contradicts ML-DSA’s assumed unforgeability. Furthermore, the inclusion of key version, authorization status, and timestamp in the signed message reduces context confusion and replay risks.
**Claim 3. ML-KEM-512 protected relay reduces relay-level metadata visibility.**

At the protected relay layer, an ML-KEM-512-based session key is established with the relay. This session key is used to protect transaction metadata with AEAD. Thus, the relay/mempool observer sees protected ciphertext instead of plaintext metadata.

**Justification:** ML-KEM is a module-lattice-based key encapsulation standard [8]. The security of the Kyber/ML-KEM structure is based on MLWE-based hardness assumptions and CCA-secure KEM transformation [25]. When used with AEAD protection, it becomes difficult to openly observe transaction metadata on the relay path. This structure reduces relay visibility and public-key exposure factors in the QES model.
**Claim 4. Threshold-based authorization reduces single-signer dependency.**

In the proposed framework, the transaction forwarding decision is not made by a single gateway or a single signer. The transaction is transferred to the protected relay layer only when at least (**t**) valid authorizer approvals are obtained.

**Justification:** This structure is used not as a new threshold signature scheme, but as a transaction-level authorization rule. Nevertheless, the security effect is clear: compromising a single signer or gateway is not sufficient for transaction authorization unless the threshold condition is met. Thus, the risks of single-signer dependency and a compromised single gateway are reduced.
**Claim 5. EBSC preserves auditability while reducing storage-based blockchain overhead.**

EBSC does not write raw sensor data, ML-DSA signature value, or ML-KEM ciphertext output directly to contract storage. Instead, it publishes compact exposure-aware metadata fields such as commitment, key version, authorization status, relay mode, QES level, and timestamp as events.

**Justification:** In blockchain-based IoT systems, storage overhead and gas cost are significant limiting factors [19,20,21]. EBSC reduces persistent storage writing while providing audit trails. The experimental results show that EBSC achieves an approximately 80.59% gas reduction compared to the StorageAudit structure. This result demonstrates that auditability is preserved while blockchain overhead is reduced with an event-based audit mechanism.
**Claim 6. QES enables transaction-level CRQC-aware exposure quantification.**

QES does not reduce transaction security assessment solely to the signature verification result. It evaluates public-key exposure, relay/mempool visibility, key reuse, signature vulnerability, migration readiness, and signer centralization factors together.

**Justification:** CRQC-aware transaction risk does not depend solely on the signature algorithm used. Where and for how long the public key remains visible, the extent to which metadata is exposed in the relay/mempool environment, the degree of key reuse, and transaction approval centralization also affect the attack surface value [6,13,14]. QES combines these factors under a single measurable score, making different transaction security scenarios comparable.

### 7.5. CRQC-Aware Exposure Reduction

The main security contribution of the proposed framework is the reduction in CRQC-aware exposure throughout the transaction workflow. In the classical open transaction scenario, the public key is openly visible, relay/mempool exposure is high, there is a risk of key reuse, the signature mechanism relies on classical cryptography, and transaction approval may be concentrated on a single signer or gateway. Under this worst-case component assignment, the QES value is computed as 100, which corresponds to the critical exposure level.

The ML-DSA-only scenario reduces the signature-level quantum vulnerability. However, relay visibility, public-key exposure, key reuse, and single-signer dependency may persist. Therefore, the QES value remains at 64.4. This result shows that simply replacing the classical signature algorithm with a post-quantum signature mechanism does not eliminate transaction-level exposure risk.

In the ML-DSA + protected relay scenario, relay visibility and metadata exposure are reduced. In this case, the QES value drops to 32.4. In the full framework scenario, ML-DSA authentication, ML-KEM-512 protected relay, threshold authorization, key lifecycle metadata, and EBSC audit are used together. Under the full-framework component assignment, the scenario-based QES value decreases to 4. This result shows that post-quantum security requires not only the replacement of cryptographic primitives but also exposure-minimization design at the transaction workflow level.

### 7.6. Conditional Attack Success Probability Interpretation

The probability of a successful attack is evaluated based on the conditions under which an adversary can exploit the transaction workflow of the proposed framework. This probability is associated with the adversary’s ability to observe the public key, relay layer, mempool, or audit metadata fields; exploit the employed signature mechanism; bypass the transaction authorization layer; benefit from key reuse or long-lived identity exposure; and obtain sufficient information advantage within the transaction workflow. QES is used as a complementary indicator for assessing the probability of a successful attack by quantifying the CRQC-aware exposure level within this transaction workflow.

Under the assumption of a CRQC-capable adversary, the conditional attack success probability for a transaction can be expressed via the following interpretive decomposition:

(26) $$P_{\mathrm{succ}}\left(tx_{i}|A_{\mathrm{CRQC}}\right)\approx P_{\mathrm{obs}}\left(tx_{i}\right)\cdot P_{\mathrm{sig}}\left(tx_{i}\right)\cdot P_{\mathrm{auth}}\left(tx_{i}\right)\cdot P_{\mathrm{reuse}}\left(tx_{i}\right).$$

where $P_{\mathrm{succ}}\left(tx_{i}|A_{\mathrm{CRQC}}\right)$ denotes the probability of a successful attack against transaction $tx_{i}$ under the CRQC-capable adversary assumption. $P_{\mathrm{obs}}\left(tx_{i}\right)$ denotes the probability that the adversary can observe useful transaction-related information, including public-key, relay-layer, mempool, or audit metadata fields. This term is reduced by protected relay communication and metadata minimization. $P_{\mathrm{sig}}\left(tx_{i}\right)$ denotes the probability that the employed signature mechanism can be exploited by a CRQC-capable adversary. In a classical-signature setting, this term is considered high because the security of ECDSA-like mechanisms is affected by sufficiently capable quantum attacks. In the ML-DSA-based setting, this term is considered negligible under the post-quantum security assumptions of the selected parameter set. $P_{\mathrm{auth}}\left(tx_{i}\right)$ denotes the probability of bypassing the transaction authorization layer. $P_{\mathrm{reuse}}\left(tx_{i}\right)$ denotes the probability that key reuse or long-lived identity exposure provides the adversary with additional exploitable information.

In a single-signer transaction flow, $P_{\mathrm{auth}}\left(tx_{i}\right)$ depends on the probability of compromising the relevant gateway or signer component. When threshold authorization is used, the attacker needs to compromise at least t authorizing nodes to pass the transaction through the authorization layer. If the independent compromise probability of each authorizing node is considered as $p_{c}$, then the authorization bypass probability for a t-of-n authorization structure is modeled as follows:

(27) Pauth=∑k=tnnkpck(1−pc)n−k.

In this expression, nk denotes the number of possible combinations for selecting k compromised authorizing nodes out of n nodes. The term $p_{c}^{k}$ denotes the probability that k authorizing nodes are compromised, while ${\left(1-p_{c}\right)}^{n-k}$ represents the probability that the remaining n−k nodes are not compromised. Therefore, $P_{a}uth$ represents the probability that the adversary compromises at least t authorizing nodes and bypasses the threshold authorization layer. This shows that threshold authorization reduces single-signer dependency and decreases the probability of a successful attack at the authorization stage.

This conditional probability interpretation explains how the framework components affect the probability of a successful attack. In the proposed framework, $P_{obs}$ is reduced by protected relay communication and metadata minimization. $P_{sig}$ is reduced by ML-DSA-based post-quantum authentication. $P_{auth}$ is reduced by threshold authorization. $P_{reuse}$ is reduced by key lifecycle metadata control. The actual numerical probability of a successful attack depends on the adversary’s CRQC capability, network observation capability, gateway-compromise probability, relay visibility, key reuse policy, and deployment conditions.

## 8. Conclusions

In this paper, we presented a CRQC-aware and exposure-minimizing blockchain transaction framework for next-generation sensor networks. We designed the proposed framework to address blockchain transaction security through the transaction-level CRQC exposure surface and to reduce quantum-induced exposure across the transaction workflow. In this context, we reduced on-chain sensor data exposure through hash-based commitments, provided post-quantum transaction authentication with ML-DSA, limited relay/mempool visibility through ML-KEM-based protected relay communication, reduced single-signer dependency through threshold-based authorization, and enabled low-cost auditability through EBSC. We used QES to quantitatively analyze the CRQC-aware transaction attack surface beyond the binary result of signature verification. We analyzed the proposed framework through a structured claim-based security analysis, demonstrating its ability to mitigate transaction-level CRQC exposure vectors under standard post-quantum security assumptions. Moreover, our experimental results on a real DPV/ECS electrochemical toxicity sensor use case validate that the proposed framework achieves efficient edge-side transaction processing, low blockchain audit overhead under full on-chain audit recording, stable behavior under controlled latency/jitter profiles, and measurable CRQC-aware exposure reduction up to the 10,000-transaction scale.

For future work, we plan to extend the current threshold-based authorization layer with post-quantum threshold signature mechanisms and validate the framework in constrained sensor, distributed edge, and private blockchain environments.

## Figures and Tables

**Table 1:** Qualitative comparison with related studies.

Study	Main Focus	PQC	IoT	EM	TA	PR	LCA
Allende et al. [15]	Quantum-resistant blockchain migration	Yes	Limited	Partial	No	Partial	No
Puneyani and Bhat [1]	Quantum-resistant blockchain transaction signing	Yes	Limited	No	No	No	Limited
Castiglione et al. [2]	Low-cost IoT device integration with PQ blockchain	Yes	Strong	No	No	No	No
Hao and Wu [3]	Edge-assisted PQ blockchain verification	Yes	Strong	Partial	No	No	No
Alanazi et al. [4]	Zero-trust IoT with blockchain, PQC, and IPFS	Yes	Strong	No	Partial	No	Partial
Liu et al. [20]	Lightweight blockchain for Industrial IoT	No	Strong	No	No	No	No
Garba [21]	Blockchain-based lightweight certificate authentication	No	Strong	No	No	No	Partial
**Proposed framework**	**CRQC-aware sensor-to-blockchain transaction security**	**Yes**	**Strong**	**Yes**	**Yes**	**Yes**	**Yes**

Note: Bold text denotes the proposed framework row. PQC: Post-quantum cryptography support; IoT: IoT/sensor support; EM: exposure model; TA: threshold authorization; PR: protected relay; LCA: low-cost audit. “Yes” indicates that the feature is explicitly supported; “No” indicates that it is not addressed; “Partial” indicates that the feature is only partially covered; “Limited” indicates a restricted or indirect treatment; and “Strong” indicates that the feature is central to the study.

**Table 2:** Notation used in this study.

Symbol	Description
$S_{i}$	The *i*-th sensor node.
*G*	Edge gateway.
*R*	Protected relay.
BC	Blockchain network.
EBSC	Event-based smart contract.
$d_{i}$	Sensor datum.
$ts_{i}$	Timestamp.
H(·)	Cryptographic hash function.
$h_{i}$	Sensor data hash.
$\left(pk_{i},sk_{i}\right)$	Public/private key pair of the *i*-th sensor node.
$pkHash_{i}$	Public-key hash.
$kv_{i}$	Key version.
$policy_{i}$	Transaction or audit policy.
$com_{i}$	Transaction commitment.
$m_{i}$	Message to be signed using ML-DSA.
$\sigma_{i}$	ML-DSA signature.
$tx_{i}$	Blockchain transaction object.
$V_{j}$	The *j*-th authorizing edge node.
$a_{j}$	Approval bit of the *j*-th authorizing node.
*t*	Minimum approval threshold.
*n*	Number of authorizing nodes.
$Auth_{i}$	Threshold authorization result.
$\left(pk_{R},sk_{R}\right)$	Relay public key and decapsulation key material.
$ct_{R}$	ML-KEM ciphertext.
$K_{R}$	Relay session key.
$M_{i}$	Transaction metadata to be transferred to the protected relay.
$AD_{i}$	Associated data for authenticated encryption with associated data (AEAD).
$C_{i}$	Encrypted transaction metadata.
$relayMode_{i}$	Relay transmission mode.
$qes_{i}$	Quantum Exposure Score value.
$E_{pk}$	Public-key exposure level.
$T_{relay}$	Relay/mempool exposure duration.
$R_{reuse}$	Key reuse indicator.
$A_{sig}$	Quantum vulnerability of the signature mechanism.
$M_{pq}$	Post-quantum migration readiness.
$S_{central}$	Single-signer or single-gateway dependency.

**Table 3:** Trust assumptions and compromise scope of the proposed framework.

Entity/Component	Trust Assumption	Compromise Impact	Remaining Security Properties
Sensor node	Generates sensor data and transmits it to the edge gateway through CoAP.	A compromised sensor node may submit incorrect or manipulated measurements.	Commitment binding and EBSC auditability remain available for the submitted data representation; physical measurement correctness is outside the cryptographic guarantee.
Edge gateway	Generates commitments, computes QES, performs ML-DSA-based transaction authentication, and prepares relay metadata.	A compromised gateway may prepare an invalid, policy-violating, or selectively disclosed transaction.	Threshold authorization can prevent forwarding if fewer than *t* authorizers approve the transaction. EBSC records only compact audit metadata.
Authorizing edge nodes	Validate the transaction commitment according to the policy and generate approval bits.	If fewer than *t* authorizers are compromised, the adversary cannot satisfy the authorization rule.	The *t*-of-*n* authorization rule preserves distributed approval and reduces single-signer dependency.
Threshold authorization layer	Requires at least *t* approvals before forwarding the transaction to the protected relay and EBSC layers.	If *t* or more authorizers are compromised, the authorization layer can be bypassed.	ML-DSA verification, commitment binding, protected relay processing, and EBSC auditability remain separate security checks.
Protected relay	Reduces readable exposure of transaction metadata during relay or mempool propagation.	Relay compromise can weaken metadata confidentiality and increase relay-level visibility.	Raw sensor data are not stored on-chain, and EBSC continues to publish only compact audit metadata.
Blockchain and EBSC	Provide append-only audit events for commitments and exposure-related metadata.	Blockchain consensus compromise is outside the evaluated scope; excessive metadata exposure may increase linkage risk.	Commitment-based auditability, key-version visibility, authorization status, relay mode, QES value, and timestamp remain available for audit.

**Table 4:** QES component weights and assignment rationale.

Component	Symbol	Weight	Rationale
Public-key exposure	$E_{pk}$	0.22	Directly enlarges the CRQC attack window.
Relay/mempool exposure duration	$T_{\mathrm{relay},\mathrm{norm}}$	0.20	Increases transaction visibility during propagation.
Key reuse risk	$R_{\mathrm{reuse}}$	0.14	Increases cumulative exposure and linkability.
Signature vulnerability	$A_{\mathrm{sig}}$	0.20	Captures classical signature vulnerability to CRQC attacks.
PQ migration readiness	$M_{\mathrm{pq}}$	0.14	Acts as a mitigating factor through $\left(1-M_{\mathrm{pq}}\right)$.
Signer centralization	$S_{\mathrm{central}}$	0.10	Captures single-signer or -gateway dependency.

**Table 5:** QES exposure level classification.

QES Range	Exposure Level
0≤QES<25	Low exposure
25≤QES<50	Moderate exposure
50≤QES<75	High exposure
75≤QES≤100	Critical exposure

**Table 6:** Cryptographic operation times by scale.

Scale	Txs.	ML-KEM Encaps. (ms)	ML-KEM Decaps. (ms)	ML-DSA Sign (ms)	ML-DSA Verify (ms)	Th. Collect (ms)	Th. Verify (ms)	AEAD Enc. (ms)	AEAD Dec. (ms)	Commit. (ms)
100	300	0.140	0.163	0.264	0.070	0.754	0.216	0.030	0.011	0.003
500	1500	0.128	0.151	0.246	0.070	0.749	0.214	0.019	0.009	0.002
1000	3000	0.126	0.151	0.251	0.070	0.737	0.215	0.017	0.008	0.002
2500	7500	0.124	0.149	0.245	0.070	0.744	0.213	0.015	0.007	0.002
5000	15,000	0.125	0.151	0.249	0.071	0.755	0.216	0.014	0.007	0.002
10,000	30,000	0.126	0.151	0.248	0.071	0.755	0.217	0.014	0.007	0.002

Note: Txs.: Transactions; Th.: threshold; Commit.: commitment computation.

**Table 7:** Latency breakdown by scale.

Scale	Txs.	CoAP Reassembly	Hash + Commitment	Threshold	ML-DSA Total	Protected Relay	Off-Chain Processing
100	300	0.339	0.066	0.974	0.347	0.477	2.202
500	1500	0.223	0.042	0.965	0.325	0.406	1.960
1000	3000	0.207	0.038	0.953	0.329	0.391	1.918
2500	7500	0.192	0.035	0.958	0.322	0.380	1.887
5000	15,000	0.180	0.035	0.971	0.326	0.383	1.895
10,000	30,000	0.187	0.035	0.973	0.326	0.383	1.904

**Table 8:** Full on-chain audit experiment under selected transaction scales.

Scale	Sensor tx	On-Chain tx	EBSC Avg Gas	StorageAudit Avg Gas	Gas Reduction	Avg Chain Latency (ms)	Avg Total Latency (ms)
100	100	200	28,037.32	144,465.32	80.59%	18.510	20.530
500	500	1000	28,036.38	144,464.38	80.59%	15.309	17.228
1000	1000	2000	28,035.98	144,463.98	80.59%	15.054	16.890
2500	2500	5000	28,035.76	144,463.76	80.59%	14.631	16.491
5000	5000	10,000	28,036.05	144,464.05	80.59%	14.635	16.507
10,000	10,000	20,000	28,035.94	144,463.94	80.59%	15.335	17.257

**Table 9:** Full on-chain latency/jitter-aware audit experiment at 10,000 sensor transactions.

Profile	Delay Range (ms)	Avg Simulated Delay (ms)	On-Chain tx	Avg Total Latency (ms)	Avg Total with Network (ms)	Latency Increase (ms)
lat10-jit5	10–15	12.504	20,000	27.892	40.396	12.504
lat50-jit20	50–70	59.935	20,000	39.490	99.424	59.934

Note: lat denotes the base simulated communication delay, and jit denotes the jitter range added to the base delay.

**Table 10:** QES scenario comparison.

Scenario	$E_{\mathrm{pk}}$	$T_{\mathrm{relay},\mathrm{norm}}$	$R_{\mathrm{reuse}}$	$A_{\mathrm{sig}}$	$M_{\mathrm{pq}}$	$S_{\mathrm{central}}$	QES	Exposure Level
Classical open	1	1	1	1	0	1	100	Critical exposure
ML-DSA only	0.8	1	0.7	0	0.5	1	64.4	High exposure
ML-DSA + protected relay	0.3	0.3	0.5	0	0.8	1	32.4	Moderate exposure
Full framework	0	0.2	0	0	1	0	4	Low exposure

**Table 11:** QES sensitivity analysis under alternative weight settings.

Scenario	Baseline	Critical +10%	Critical −10%	Balanced
Classical open	100.00	100.00	100.00	100.00
ML-DSA only	64.40	64.18	64.65	66.67
ML-DSA + protected relay	32.40	31.69	33.20	38.33
Full framework	4.00	4.14	3.84	3.33

**Table 12:** Same-environment ablation and baseline comparison.

Configuration	Signature	Relay	Threshold	Avg Total Latency (ms)	CoAP Payload (B)	EBSC/StorageAudit Avg Gas	QES
Classical open baseline	ECDSA	Open	No	15.691	932.4	28,036.29/144,464.29	100.0
ML-DSA only	ML-DSA	Open	No	15.223	928.4	28,036.00/144,464.00	64.4
ML-DSA + protected relay	ML-DSA	Protected	No	16.977	939.4	28,047.57/144,475.57	32.4
ML-DSA + threshold, open relay	ML-DSA	Open	Yes	17.523	944.4	28,036.00/144,464.00	40.4
Full framework	ML-DSA	Protected	Yes	17.164	932.4	28,035.89/144,463.89	4.0

Note: Average gas values are reported per audit record. EBSC and StorageAudit were executed for each configuration under the same Ganache environment.

**Table 13:** Metric-level quantitative comparison with related studies.

Study	Platform/Context	Quantitative Metrics
Puneyani and Bhat [1]	Dilithium-based quantum-resistant blockchain	Latency +20%; hybrid latency ≈ 1.2 ms/tx; throughput ≈ 400 tx/s.
Castiglione et al. [2]	ESP32-based low-cost IoT device using Dilithium-5	Key generation ≈ 70.30 ms; signing ≈ 316.39–394.68 ms; verification ≈ 73.23 ms.
Alanazi et al. [4]	Zero-trust IoT with blockchain, PQC, IPFS, and anomaly detection	Latency reduction up to 35%; throughput increase 40%; on-chain storage reduction 70%; anomaly detection accuracy > 90%.
Si et al. [32]	Smart contract-based continuous dual-offline cryptocurrency payment	Gas decreases from ≈32,000 to ≈29,000 per transaction; execution time decreases from 19 ms to 12–14 ms.
**Proposed framework**	**Node.js edge gateway, CoAP, PQClean/WASM, Ganache, real DPV/ECS sensor data**	**ML-KEM encap. 0.124–0.140 ms; decap. 0.149–0.163 ms; ML-DSA sign 0.245–0.264 ms; verify 0.070–0.071 ms; off-chain processing 1.887–2.202 ms; EBSC gas reduction 80.59%; QES 100 → 4.**

Note: Bold text denotes the proposed framework row.

## Data Availability

The sensor data used in this study were obtained from previously collected DPV/ECS electrochemical toxicity measurements and were used with permission for the experimental evaluation of the proposed framework. The original contributions and experimental results generated in this study are included in this paper. Further inquiries can be directed to the corresponding author.

## Abbreviations

The following abbreviations are used in this manuscript:

ABI	Application Binary Interface
AEAD	Authenticated Encryption with Associated Data
CoAP	Constrained Application Protocol
CRQC	Cryptographically Relevant Quantum Computer
DPV	Differential Pulse Voltammetry
EBSC	Event-Based Smart Contract
ECS	Electrochemical Sensor
IoT	Internet of Things
KEM	Key Encapsulation Mechanism
ML-DSA	Module-Lattice-Based Digital Signature Algorithm
ML-KEM	Module-Lattice-Based Key Encapsulation Mechanism
PQC	Post-Quantum Cryptography
QES	Quantum Exposure Score
WASM	WebAssembly
